# A TonB-Like Protein, SjdR, Is Involved in the Structural Definition of the Intercellular Septa in the Heterocyst-Forming Cyanobacterium *Anabaena*

**DOI:** 10.1128/mBio.00483-21

**Published:** 2021-06-08

**Authors:** Hannah Schätzle, Sergio Arévalo, Enrique Flores, Enrico Schleiff

**Affiliations:** a Institute for Molecular Biosciences, Goethe University Frankfurt, Frankfurt am Main, Germany; b FIERCE, Goethe University Frankfurt, Frankfurt am Main, Germany; c Instituto de Bioquímica Vegetal y Fotosíntesis, CSIC and Universidad de Sevilla, Seville, Spain; d Buchmann Institute for Molecular Life Sciences, Frankfurt am Main, Germany; e Frankfurt Institute for Advanced Studies, Frankfurt am Main, Germany; University of Nebraska—Lincoln

**Keywords:** cyanobacteria, TonB-like proteins, cell wall, heterocyst, peptidoglycan, cyanobacteria

## Abstract

Cyanobacteria are photosynthetic organisms with a Gram-negative envelope structure. Certain filamentous species such as *Anabaena* sp. strain PCC 7120 can fix dinitrogen upon depletion of combined nitrogen. Because the nitrogen-fixing enzyme, nitrogenase, is oxygen sensitive, photosynthesis and nitrogen fixation are spatially separated in *Anabaena*. Nitrogen fixation takes place in specialized cells called heterocysts, which differentiate from vegetative cells. During heterocyst differentiation, a microoxic environment is created by dismantling photosystem II and restructuring the cell wall. Moreover, solute exchange between the different cell types is regulated to limit oxygen influx into the heterocyst. The septal zone containing nanopores for solute exchange is constricted between heterocysts and vegetative cells, and cyanophycin plugs are located at the heterocyst poles. We identified a protein previously annotated as TonB1 that is largely conserved among cyanobacteria. A mutant of the encoding gene formed heterocysts but was impaired in diazotrophic growth. Mutant heterocysts appeared elongated and exhibited abnormal morphological features, including a reduced cyanophycin plug, an enhanced septum size, and a restricted nanopore zone in the septum. In spite of this, the intercellular transfer velocity of the fluorescent marker calcein was increased in the mutant compared to the wild type. Thus, the protein is required for proper formation of septal structures, expanding our emerging understanding of *Anabaena* peptidoglycan plasticity and intercellular solute exchange, and is therefore renamed SjdR (septal junction disk regulator). Notably, calcium supplementation compensated for the impaired diazotrophic growth and alterations in septal peptidoglycan in the *sjdR* mutant, emphasizing the importance of calcium for cell wall structure.

## INTRODUCTION

Cyanobacteria like *Anabaena* sp. strain PCC 7120 (hereinafter *Anabaena*) perform oxygenic photosynthesis and have a Gram-negative type of cell wall. They contain a plasma membrane (PM) and an outer membrane (OM) separated by a peptidoglycan (PG) mesh in the periplasm (1, 2). *Anabaena* is a multicellular organism in which one filament may comprise hundreds of cells. Specialized cells named heterocysts are formed from vegetative cells upon nitrogen starvation (3). In contrast to the vegetative cells that perform oxygenic photosynthesis, oxygen production in heterocysts is avoided due to metabolic adaptations such as photosystem II activity shutdown (4). This, in combination with the formation of additional heterocyst-specific envelope layers, allows the formation of a microoxic environment, which in turn is required for proper nitrogenase activity (5, 6). The nitrogenase catalyzes the fixation of dinitrogen into ammonium. In *Anabaena*, the nitrogenase structural genes are exclusively expressed in heterocysts (7). The spatial separation between carbon and nitrogen fixation results in a mutual dependency of vegetative cells and heterocysts and a requirement for metabolite exchange between the cells. For this, septal junctions exist that allow the intercellular diffusion of certain compounds.

The perforations in the septal PG between adjacent cells are called nanopores (2, 8). SepJ (FraG), FraC and FraD were identified as proteinaceous compounds related to the septal junctions (9, 10). The knockout of either *sepJ* or both *fraC* and *fraD* in *Anabaena* affects intercellular molecular transfer of different fluorescent markers, although to a different extent (8, 11, 12). In addition, SjcF1 was reported to connect PG and septal junction complexes influencing nanopore formation (13). The cell wall amidases AmiC1 and AmiC2 are thought to drill the nanopores in the PG (14). AmiC1 is important for both nanopore formation and septal junction complex integrity, since it influences the localization of septal proteins (14). The deletion of septal or nanopore-related proteins affects diazotrophic growth and filament integrity in *Anabaena*. For example, the inactivation of *sepJ* or *amiC1* impairs heterocyst differentiation in the respective mutant strain (9, 14, 15). Deletion of *sepJ* or the *fraC* and/or *fraD* genes moreover leads to extensive filament fragmentation (9, 10, 12). In addition, a relation between septal proteins and the regulation of cell division and growth can be drawn. It has been reported that SepJ interacts with the divisome proteins ZipN (16) and FtsQ (17) as well as with the two elongasome-associated proteins ZicK and ZacK (18). Divisomal and elongasomal components function in PG formation and thereby in the determination of cell shape (16, 18–20). Whereas the divisome coordinates cell constriction and division, the elongasome incorporates PG along the sidewall and mediates longitudinal cell extension (21, 22). Indeed, inactivation of the *mreB*, *mreC*, and *mreD* genes encoding elongasome components leads to increased septal width and septal PG incorporation (23).

In Gram-negative bacteria, TonB-dependent transport represents a conserved mechanism that mediates the active translocation of compounds across the OM. Siderophores, carbohydrates, vitamin B_12_, or nickel are substrates for TonB-dependent transporters (TBDT) (24–27). In contrast to the septal junction complex, which is restricted to filamentous cyanobacteria, approximately two thirds of all Gram-negative bacteria bear genes encoding TonB-dependent transport system components (28). The details of the TonB-dependent transport processes in Gram-negative bacteria are partly known. The extracellular binding of the substrate to the TBDT induces the exposition of the TonB box, a semiconserved motif in the N-terminal part of the TBDT (29). The TonB box has a high affinity to bind the C terminus of the PM-embedded TonB protein (30, 31). TonB proteins consist of a conserved N-terminal alpha-helical membrane anchor, a flexible periplasmic linker region that is often proline rich, and a C-terminal TonB box interaction domain that bears a conserved secondary structure (28). In general, these proteins lack a cytoplasmic domain. ExbB and ExbD harvest the energy of the proton motive force and transmit it to TonB that transduces it to the TBDT. By this mechanism, conformational changes are induced in the transporter, which allows the entrance of the substrate into the periplasmic space (28, 32–34).

Regarding cyanobacteria, the TonB-dependent siderophore transport network has been partially characterized in *Synechocystis* sp. strain PCC 6803 (35–39) and *Anabaena* (40–43). In the *Anabaena* genome, 22 TBDT-encoding genes have been identified, but only 4 genes encode putative TonB proteins, referred to as TonB1 to TonB4 (44). The four presumptive TonB proteins bear disparate structural features and their functional properties have been poorly characterized. *Anabaena* TonB3 (encoded by *all5036*) comprises conserved motifs characteristic for TonB proteins, and its involvement in the transport of ferric siderophores has been shown (44). TonB2 (encoded by *all3585*) and TonB4 (encoded by *alr5329*) are shortened in the C-terminal part compared to TonB3, and TonB1 (encoded by *alr0248* and here renamed SjdR) exhibits the most exceptional domain structure. The latter protein bears an N-terminal extension that is putatively exposed to the cytosol, which is not typically found in TonB proteins. Further, it contains a C-terminal truncation resulting in an incomplete TonB box binding motif (44). Considering this, it remains questionable whether this protein can bridge the periplasm in order to interact with TBDT. The expression of *alr0248* was not enhanced when *Anabaena* was grown in iron-free medium. However, induction of *alr0248* was observed when cells were grown in medium without a combined nitrogen source (44), a condition that in *Anabaena* elicits production of heterocysts. Thus, the available expression data for *alr0248* and the structural composition of the encoded protein raise concerns regarding an involvement of this protein in energizing TBDT-mediated transport.

Consequently, we approached the function of the protein annotated as TonB1 in *Anabaena* by analyzing a mutant strain with a mutant gene. This strain was retarded in growth under diazotrophic conditions due to a low nitrogenase activity, and the mutant heterocysts appeared abnormally elongated. Moreover, the septal PG arrangement and the nanopore zone diameter in the mutant were altered compared to the wild type, which in turn influenced the rate of intercellular molecular diffusion in the mutant strain. This implies that the protein influences PG morphology with an important role in the ultrastructure of the intercellular septa, and therefore, we rename it SjdR (septal junction disk regulator).

## RESULTS

### SjdR is conserved in cyanobacteria.

**FIG 1:**
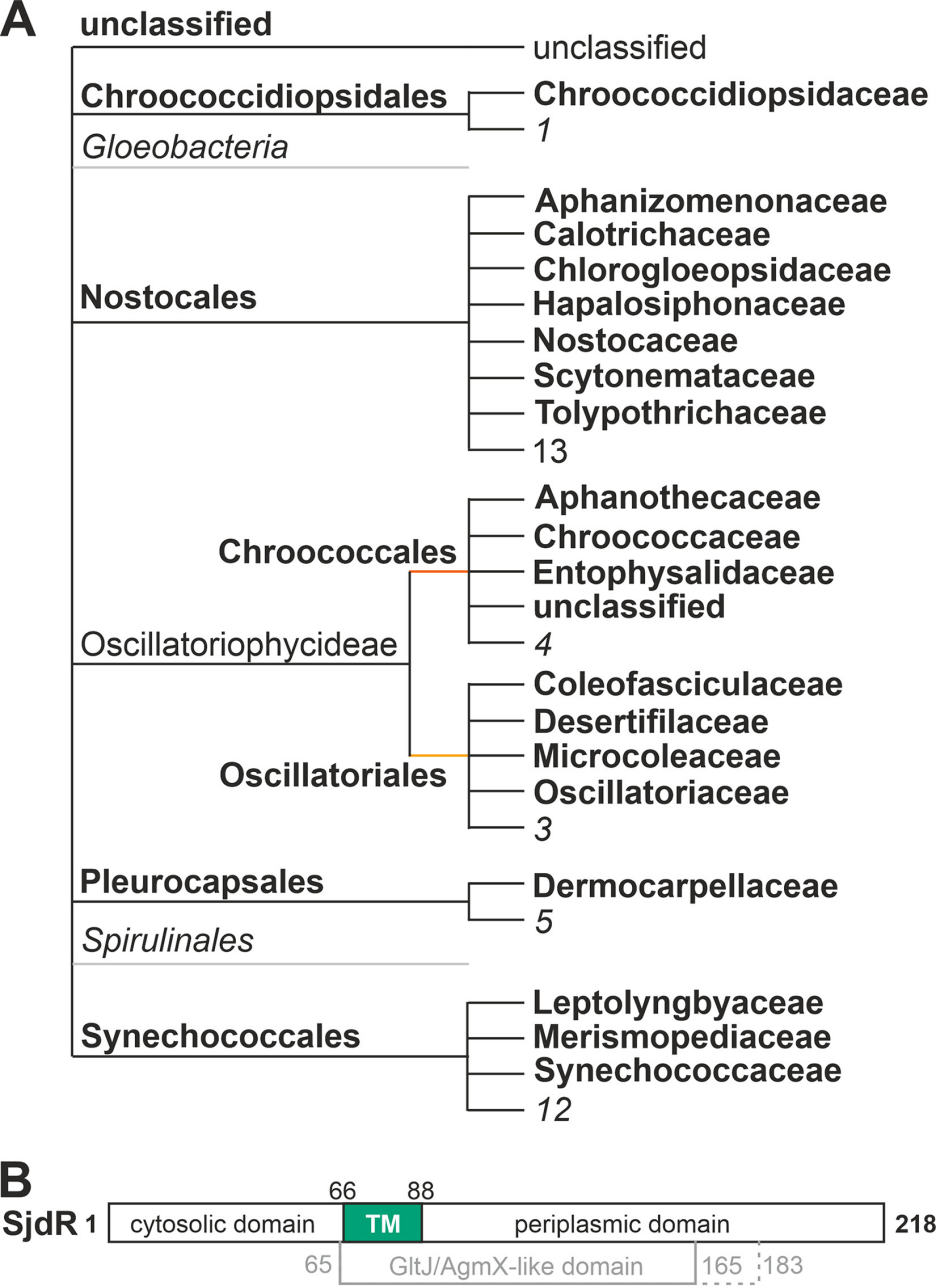
Proteins with similarity to SjdR (formerly named TonB1) from *Anabaena* in cyanobacteria. (A) The cyanobacterial order and the family in which a protein sequence with similarity to SjdR was identified is shown. The numbers of families in which no SjdR-like proteins were identified is indicated as well. (B) Domain architecture of SjdR. The position of the predicted GltJ/AgmX-like domain is shown. The solid line represents the domain deposited in the CDD database. The dashed line indicates the conserved TonB-like region identified by individual alignment. The periplasmic domain also shows homology to a cytoplasmic part of ZipA. The numbers of amino acids that delimit domains are given. TM, transmembrane domain.

The amino acid sequence of SjdR (formerly annotated as TonB1) substantially differs from those of TonB2, TonB3, and TonB4 (44), which might suggest a functional diversification. Thus, the prevalence of SjdR within the cyanobacterial phylum was analyzed. Fifty cyanobacterial SjdR-like sequences were identified in seven cyanobacterial orders and 20 families (Fig. 1A). Only in *Gloeobacteria* and *Spirulinales* could a SjdR-like sequence not be identified. A list of all cyanobacterial species where SjdR-like sequences were predicted is provided in Table S5 in the supplemental material. The high conservation of SjdR-like proteins in cyanobacteria implies a significant functionality of SjdR. Moreover, a separation to TonB3 became obvious when BLAST search analysis was performed, as the identified sequences did not overlap.

10.1128/mBio.00483-21.8 TABLE S5 Cyanobacteria with SjdR-like sequences. Download Table S5, DOCX file, 0.02 MB. Copyright © 2021 Schätzle et al. 2021 Schätzle et al. https://creativecommons.org/licenses/by/4.0/ This content is distributed under the terms of the Creative Commons Attribution 4.0 International license.

A conserved domain prediction showed that SjdR contains a cytosolically exposed N-terminal domain of about 65 amino acids, a transmembrane domain in the center of the sequence, and a predicted periplasmic domain rich in Pro (16.9%), Thr (16.2%), Ser (13.8%), and Gln (13.1%; Fig. 1B). This domain shows homology to parts of ZipA, which is involved in cell division by stabilizing the FtsZ ring and anchoring it to the plasma membrane in different bacteria (45). However, the transmembrane domain and the C-terminal domain are similar to a membrane-anchored and periplasm-exposed domain in GltJ/AgmX proteins as well (amino acids 65 to 165; Fig. 1B). These proteins are related to the so-called adventurous gliding motility that is a pilus-independent mode of motility (46). Remarkably, GltJ proteins bear a C-terminal domain similar to the TonB C terminus (47), but this region does not align to SjdR. To the best of our knowledge, no ZipA or GltJ/AgmX homolog has been discovered in cyanobacteria. Nonetheless, these observations strengthen the assumption that SjdR has function(s) that may not be related to TonB-dependent transport.

### SjdR is not involved in transport of the endogenous siderophore.

**FIG 2:**
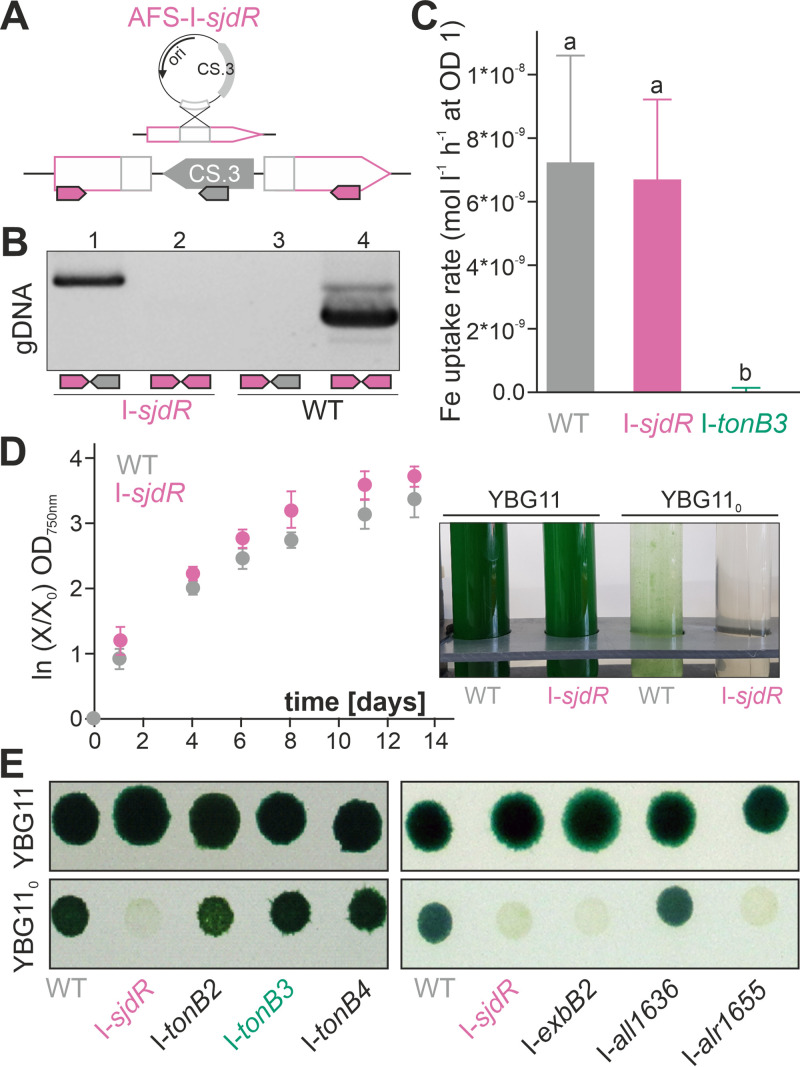
Siderophore transport capacity and diazotrophic growth defects of the *Anabaena sjdR* mutant. (A, top) The strategy of single recombination yielding plasmid insertion into the genome is illustrated. (Bottom) The genotype of the I-*sjdR* mutant is illustrated. The positioning and directionality of oligonucleotides used for the segregation analysis are indicated by arrows. (B) Confirmation of the segregation of I-*sjdR* by PCR on gDNA isolated from I-*sjdR* (lanes 1 and 2) or wild type (WT) (lanes 3 and 4) utilizing oligonucleotides indicated in panel A. (C) The means and standard deviations (error bars) of schizokinen uptake rates of wild-type (WT), I-*sjdR*, and I-*tonB3* strains are shown (*n* = 15 for WT, *n* = 6 for I-*sjdR*, *n* = 6 for I-*tonB3*). Statistical analysis was done with analysis of variance (ANOVA) and Duncan’s multiple range test: different letters above the bars indicate statistically significant differences (*P* < 0.05). (D) Growth of wild-type (gray) and I-*sjdR* (pink) strains was monitored in liquid YBG11 for the indicated times. (Right) Cultures grown for 5 days in YBG11 or YBG11_0_ were photographed. (E) Growth of the indicated mutants on plates composed of YBG11 (top) or YBG11_0_ (bottom) after spotting of 5 μl (OD_750_ of 1) is shown. Images were taken 7 (right) and 11 days (left) after spotting.

To assess the role of SjdR (previously annotated as a TonB protein) in siderophore transport, a *sjdR* single recombinant mutant was generated by plasmid insertion. The mutant strain was named AFS-I-*sjdR* (*Anabaena* sp. mutant generated in Frankfurt, Germany by the Schleiff lab by plasmid insertion; subsequently termed I-*sjdR*; Fig. 2A; Tables S1 to S3). The strain was segregated after several rounds of dilution. A PCR product of 2,041 bp was obtained with the gene-specific and plasmid-specific oligonucleotide primers on genomic DNA (gDNA) isolated from the mutant (Fig. 2B, lane 1). No fragment was obtained when gDNA from the wild-type strain was utilized as template (lane 3). The product corresponding to a wild-type locus using gene-specific oligonucleotides was obtained by PCR on gDNA from the wild type (lane 4; 1,433 bp). It should be noted that polar effects of the integration of the inactivating plasmid into the *sjdR* locus are not expected, since individual transcriptional start sites are predicted for *alr0249*, which is localized downstream of the *sjdR* open reading frame (48). Moreover, transcriptome sequencing (RNA-Seq) data imply that *sjdR* is not part of an operon but transcribed individually (49).

10.1128/mBio.00483-21.4 TABLE S1 Oligonucleotides used in this study. Download Table S1, DOCX file, 0.01 MB. Copyright © 2021 Schätzle et al. 2021 Schätzle et al. https://creativecommons.org/licenses/by/4.0/ This content is distributed under the terms of the Creative Commons Attribution 4.0 International license.

In order to examine whether SjdR is involved in energizing the transport of ferric schizokinen, short-term transport uptake measurements were conducted with radiolabeled ferric schizokinen as the substrate. Prior to the uptake measurements, the experimental cultures were depleted for iron, since schizokinen transport is enhanced in iron-starved cells (43). The starvation level was estimated by measuring the chlorophyll *a* (Chl) concentration normalized to the optical density at 750 nm (OD_750_) (Table S4). The cellular accumulation of the substrate was monitored over time in wild-type, I-*sjdR*, and AFS-I-*tonB3* (I-*tonB3* [44]) strains. An average Fe uptake rate of (6 ± 2.5) × 10^−9 ^mol liter^−1^ h^−1^ at an OD_750_ of 1 was measured for I-*sjdR*, which is comparable to the average transport rate obtained for the wild type [(7  ± 3.4) × 10^−9 ^mol liter^−1^ h^−1^ at an OD_750_ of 1 (Fig. 2C)]. In contrast, the ferric schizokinen transport was severely affected in I-*tonB3* compared to the wild type (Fig. 2C), confirming previous indications that TonB3 mediates the transport of ferric compounds (44). Therefore, schizokinen transport in *Anabaena* depends on TonB3 but not on SjdR.

10.1128/mBio.00483-21.7 TABLE S4 Starvation status of cultures utilized for schizokinen uptake measurements. Download Table S4, DOCX file, 0.01 MB. Copyright © 2021 Schätzle et al. 2021 Schätzle et al. https://creativecommons.org/licenses/by/4.0/ This content is distributed under the terms of the Creative Commons Attribution 4.0 International license.

### I-*sjdR* growth is defective under diazotrophic conditions.

Previous experiments indicated that the *sjdR* gene is expressed at higher levels in *Anabaena* after growth for 1 week under diazotrophic conditions compared to nitrate-replete conditions (44). Hence, the growth of wild-type and I-*sjdR* strains was examined under diazotrophic conditions in liquid medium and on plates (YBG11_0_ medium contains no combined nitrogen source). The growth of the two strains in standard (nitrate-containing) liquid or solid medium was used as a control (YBG11). In the presence of nitrate, the growth of both strains in liquid (Fig. 2D) or on solid medium (Fig. 2E, upper panels) was comparable. In the absence of combined nitrogen, I-*sjdR* was severely affected in growth in both liquid (Fig. 2D, right) and solid medium (Fig. 2E, lower panels, Fig. S2A). Different time points for solid and liquid growth are shown because the growth delay of I-*sjdR* becomes obvious earlier in liquid medium, in which the cells typically divide faster, than on plates.

10.1128/mBio.00483-21.2 FIG S2 Growth of the wild type and the I-*sjdR* mutant in the presence and absence of combined nitrogen. (A) Five-microliter portions of cultures with an optical density at 750 nm of 1 or 0.1 were spotted on plates containing nitrate (YBG11) or on plates without combined nitrogen source (YBG11_0_). The pictures for days 7 and 11 are taken from the same plate; the other pictures show independent plates. (B) Growth of the wild type and I-*sjdR* mutant in liquid cultures without a source of combined nitrogen with the indicated concentrations of calcium. YBG11_0_ contains a final concentration of 245 μM CaCl_2_. Download FIG S2, TIF file, 1.3 MB. Copyright © 2021 Schätzle et al. 2021 Schätzle et al. https://creativecommons.org/licenses/by/4.0/ This content is distributed under the terms of the Creative Commons Attribution 4.0 International license.

To examine the specificity of the phenotype, the behavior of I-*sjdR* in the absence of combined nitrogen was compared to the growth of the other *tonB* mutants. To control for the conditions used, growth was compared to growth of an *exbB2* mutant (*alr4587*; I-*exbB2*) and mutant strains of two unrelated genes (I-*all1636*; I-*alr1655*). In the presence of nitrate, the growth of all mutants was indistinguishable from wild-type growth (Fig. 2E, upper panels). In the absence of fixed nitrogen, the plasmid insertion mutants of *tonB2*, *tonB3*, and *tonB4* were not impaired in growth (Fig. 2E, lower panel, left). Similarly, the growth of the control mutant I-*all1636* was not affected on YBG11_0_ plates compared to the wild type (Fig. 2E, lower panel, right). In turn, I-*exbB2* and I-*alr1655* did not grow in the absence of fixed nitrogen (Fig. 2E) similar to I-*sjdR*. Hence, among mutants of the formerly annotated *tonB* genes, only the growth of the *sjdR* mutant was impaired in the absence of combined nitrogen (Fig. 2E).

### Nitrogenase activity and transcriptional analysis in the I-*sjdR* mutant strain.

**FIG 3:**
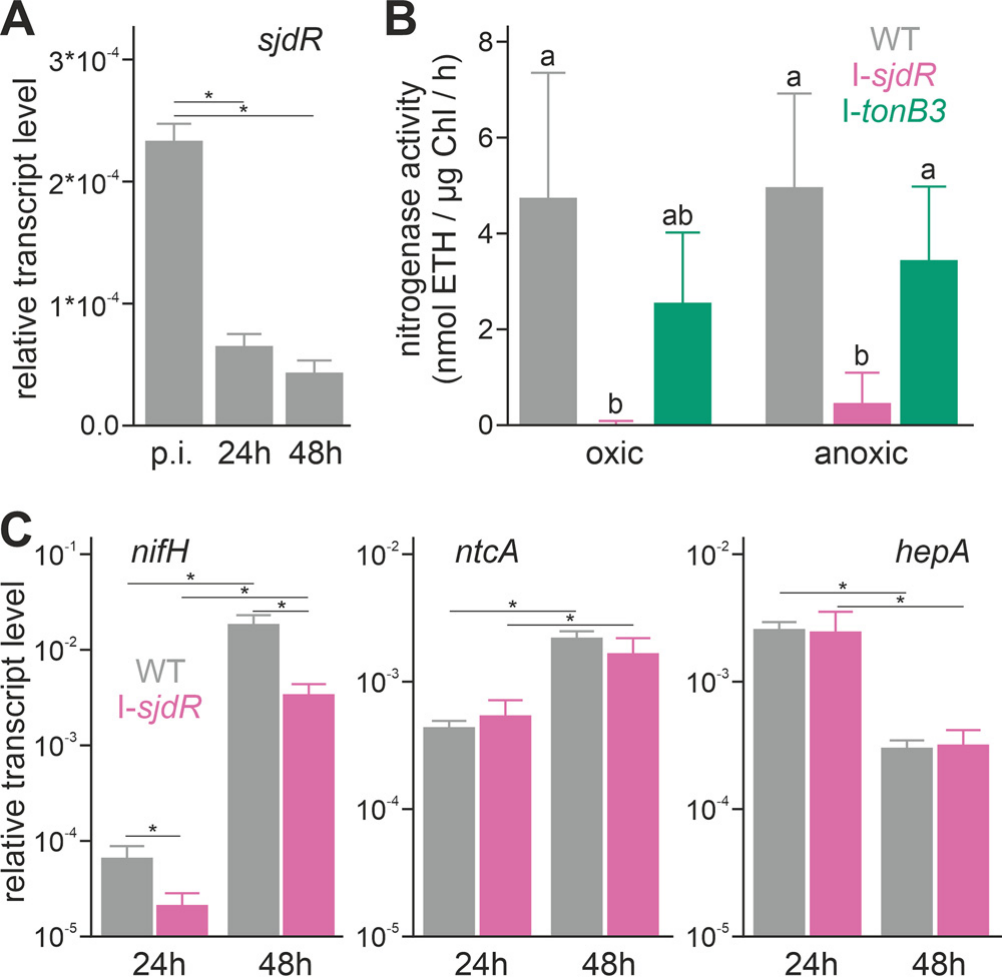
The *sjdR* mutant is impaired in nitrogenase gene expression and enzyme activity. (A) Transcript levels of *sjdR* were determined by qRT-PCR on RNA isolated from the wild type before (preinduction [p.i.]) and 24 h or 48 h after inoculation in nitrate-depleted YBG11_0_ medium. Error bars represent the standard deviations of three biological replicates per strain. Asterisks above the bars show significant differences to the p.i. sample determined with Student’s *t* test (*P* < 0.05). (B) The nitrogenase activity (in nanomoles of ethylene [ETH] per microgram of Chl per hour) was measured in wild-type (*n* = 9; gray), I-*sjdR* (*n* = 4; pink), and I-*tonB3* (*n* = 3; green) strains after 48 h of induction. Error bars represent the standard deviations of independent experiments. Statistical analysis was performed with ANOVA and Duncan’s multiple range test; bars that share the same letters are not significantly different from each other (*P* < 0.05). (C) The transcript levels of *nifH*, *ntcA*, and *hepA* were determined in wild-type (gray) and I-*sjdR* (pink) strains and normalized to expression of *rnpB*. Error bars represent the standard deviations of three biological replicates each. Significant differences are indicated by asterisks (Student’s *t* test, *P* < 0.05).

The *sjdR* mutant shows a strongly retarded growth in the absence of a source of combined nitrogen (Fig. 2), which might indicate an involvement of SjdR in heterocyst differentiation or function. The transcript levels of *sjdR* were then examined in wild-type cultures grown in YBG11_0_ medium. Cells were harvested before transfer to YBG11_0_ medium (“preinduction”) and subsequently after 24 h and 48 h of cultivation in YBG11_0_. During this time frame, heterocyst induction and maturation take place, and the expression of the nitrogenase genes peaks (50); therefore, an induction of *sjdR* expression during this stage might indicate a relation to heterocyst differentiation. The expression was analyzed by quantitative reverse transcription-PCR (qRT-PCR), and for normalization, the values were compared to those of the *rnpB* gene. The *sjdR* mRNA was less abundant in cells that were grown for 24 h or 48 h in YBG11_0_. After 24 h, only 30% of the transcript was found compared to the preinduction sample (Fig. 3A). After 48 h, the *sjdR* transcript abundance was reduced to about 20% of the preincubation sample (Fig. 3A). Consequently, it seems unlikely that SjdR actively functions in heterocyst differentiation.

In heterocysts, molecular nitrogen is fixed by the nitrogenase complex, which requires a microoxic environment to maintain functionality (6). We intended to assess to which degree the enzyme activity might be affected in I-*sjdR* after 48 h of induction, when heterocyst differentiation is completed. Therefore, the nitrogenase activity in the wild type, I-*sjdR* mutant, and I-*tonB3* mutant as control strain was measured under oxic and anoxic conditions (Fig. 3B). The nitrogenase activity in the I-*sjdR* mutant was null under oxic conditions but increased appreciably, albeit to a low level, under anoxic conditions (Fig. 3B). This suggests a Fox^−^ Fix^+^ phenotype for I-*sjdR* strain, which refers to the inability of the strain to reduce acetylene specifically under oxic conditions (51, 52). In contrast, the nitrogenase activity in the I-*tonB3* mutant was not significantly different from the wild-type activity irrespective of the conditions used (Fig. 3B).

The low enzyme activity that, nonetheless, was observed under anoxic conditions might be related to reduced transcription of the nitrogenase-encoding genes. Thus, the mRNA abundance of the gene coding for dinitrogenase reductase (*nifH*), as well as of genes related to nitrogen control (*ntcA*) and heterocyst differentiation (*hepA*) (5), was determined by qRT-PCR. RNA was isolated from wild-type and I-*sjdR* strains 24 h and 48 h after transfer to YBG11_0_ medium. The expression of *nifH* typically peaks after the heterocysts have maturated (53). Consistent with this, *nifH* transcripts were detected after 24 h, and a significant increase was observed after 48 h, both in the I-*sjdR* mutant and the wild type (Fig. 3C, left). However, compared to the wild type, the *nifH* transcript was less abundant in I-*sjdR* at both time points, indicating a transcriptional delay in the mutant, while the enzyme activity after 2 days was abolished completely. Nonetheless, the abolishment of nitrogenase function under oxic conditions in the I-*sjdR* mutant cannot be completely traced back to the moderate decrease in *nifH* transcription. In contrast, *ntcA* and *hepA* exhibited a similar transcript abundance pattern in both strains (Fig. 3C, middle and right). Thus, SjdR might not have a general impact on the regulation of heterocyst differentiation, but it seems to influence heterocyst function.

### Calcium supplementation partially recovers diazotrophic growth of the *sjdR* mutant.

In *Anabaena*, the intracellular prevalence of different metals is important for photosynthesis and diazotrophy. The nitrogenase complex possesses a FeMo cofactor, whereas the oxygen-evolving complex of photosystem II contains calcium and manganese ions (54). Thus, altered levels of metals during initial growth under nitrate-replete conditions could contribute to the impaired growth of the *sjdR* mutant upon nitrogen deprivation. Therefore, the intracellular concentration of metal ions was determined in the wild-type (data from reference 55) and I-*sjdR* strains.

**FIG 4:**
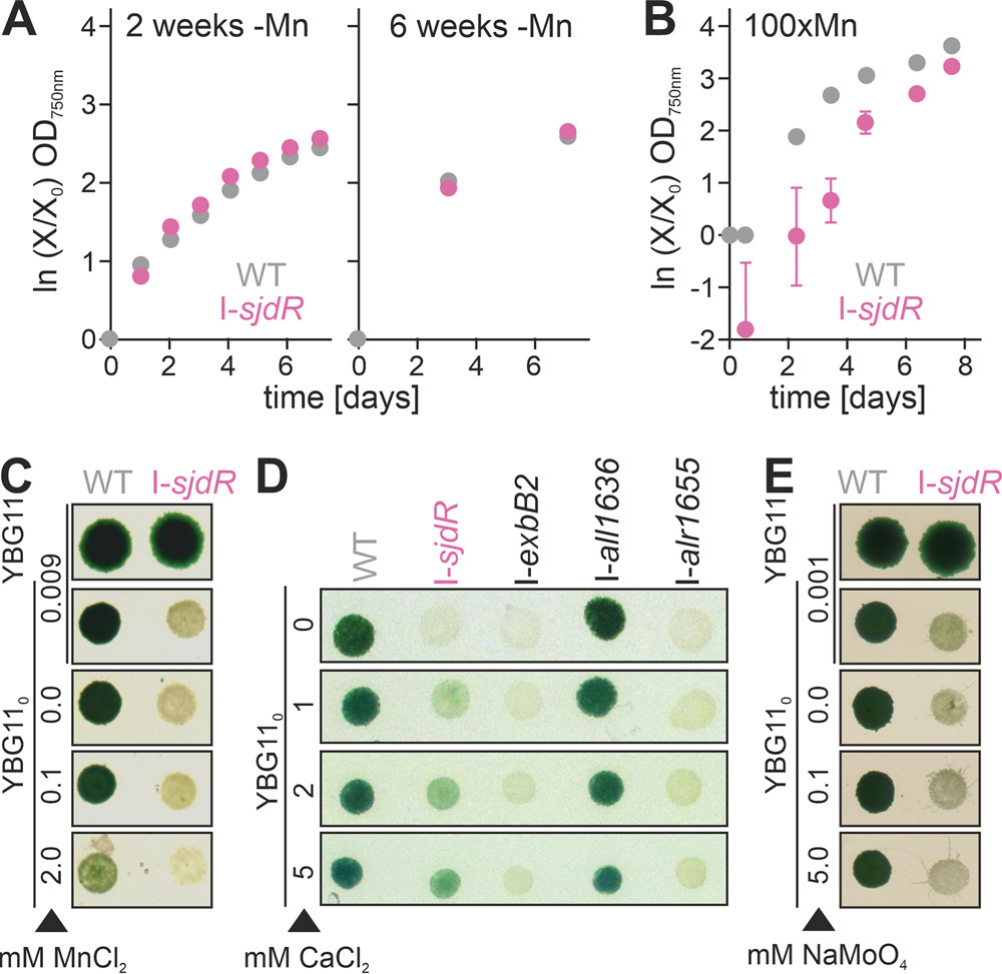
Elevated calcium concentrations stimulate the growth of the I-*sjdR* mutant strain in the absence of combined nitrogen. (A and B) Growth of wild-type (WT) (gray) and I-*sjdR* (pink) strains in YBG11 without Mn (A) or with high Mn concentrations (100 × Mn = 900 μM MnCl_2_) (B). For Mn starvation, cells were incubated 2 (A, left) or 6 (A, right) weeks in Mn-free medium with dilution to an OD_750_ of 0.05 every 7 days. The average values of ≥3 biological replicates are shown; error bars represent the standard deviations. (C to E) Growth of wild type (WT) and indicated mutants on plates composed of YBG11 or YBG11_0_ medium containing the indicated concentration of MnCl_2_ (C), CaCl_2_ (D), or Na_2_MoO_4_ (E). Standard YBG11 medium contains 9 μM MnCl_2_, 245 μM CaCl_2_, and 1 μM Na_2_MoO_4_. Cell suspensions were set to an OD_750_ of 1, 5-μl samples were spotted onto the agar plates, and the plates were incubated 7 days under culture conditions. Representative experiments are shown.

Inductively coupled plasma mass spectrometry (ICP-MS) measurements unraveled a significant increase of the calcium and molybdenum concentration, as well as a strong decrease of the manganese concentration in the I-*sjdR* mutant compared to the wild type (Table 1). The cobalt level was lower in the I-*sjdR* mutant as well but to a lesser extent than manganese. This suggests an enhanced uptake of Ca and Mo by the mutant strain, while Mn uptake is likely reduced. Consequently, the growth capacity of wild-type and mutant strains under decreased or enhanced manganese concentrations was tested. Manganese depletion did not yield a significant difference in growth between the I-*sjdR* mutant and the wild type, irrespective of the duration of starvation (Fig. 4A). Therefore, Mn limitation appears not to be problematic for *Anabaena* and I-*sjdR*. In contrast, elevated concentrations of manganese (100 × Mn = 900 μM) were increasingly detrimental to the I-*sjdR* mutant compared to the wild type (Fig. 4B). To test whether manganese affects the growth of the I-*sjdR* mutant when transferred to nitrate-free medium, manganese was either omitted or the concentration was enhanced (100 μM, 2 mM). However, the altered manganese concentrations did not recover the diazotrophic growth of the I-*sjdR* mutant (Fig. 4C). The measurements of the intracellular zinc concentration showed a high degree of variation in the *sjdR* mutant strain (Table 1), and therefore, alterations in the zinc content between the two strains were omitted from the discussion.

**TABLE 1:** Metal concentration in wild-type *Anabaena* and the I-*sjdR* mutant strain^*a*^

Medium and element	Wild-type		I-*sjdR*		I-*sjdR*/WT
	Atoms/OD	Atoms/cell^*b*^	Atoms/OD	Atoms/cell^*b*^	
YBG11					
Ca	(6.1 ± 0.7) × 10^14^	(1.2 ± 0.2) × 10^7^	(1.1 ± 0.2) × 10^15^	(2.6 ± 0.9) × 10^7^	***2.1***
Mn	(5.1 ± 0.2) × 10^14^	(1.0 ± 0.3) × 10^7^	(1.6 ± 0.1) × 10^14^	(3.5 ± 0.9) × 10^6^	***0.3***
Mg	(5.0 ± 0.4) × 10^15^	(1.0 ± 0.2) × 10^8^	(4.6 ± 1.8) × 10^15^	(1.0 ± 0.3) × 10^8^	0.9
Zn	(5.4 ± 0.3) × 10^13^	(1.1 ± 0.3) × 10^6^	(1.2 ± 1.4) × 10^14^	(2.7 ± 3.3) × 10^6^	2.2
Mo	(6.4 ± 0.2) × 10^13^	(1.3 ± 0.3) × 10^6^	(1.1 ± 0.1) × 10^14^	(2.4 ± 0.7) × 10^6^	***1.7***
Co	(1.8 ± 0.1) × 10^13^	(3.7 ± 0.8) × 10^5^	(1.5 ± 0.1) × 10^13^	(3.4 ± 1.0) × 10^5^	***0.8***
Cu	(5.1 ± 0.5) × 10^13^	(1.1 ± 0.3) × 10^6^	(5.1 ± 0.4) × 10^13^	(1.1 ± 0.3) × 10^6^	1.0
Ni	(0.4 ± 0.2) × 10^13^	(0.9 ± 0.4) × 10^5^	(0.4 ± 0.1) × 10^13^	(0.9 ± 0.2) × 10^5^	0.9

a Data are presented as the number of atoms/OD_750_ and the number of atoms/cell. The ratio of the metal content in the wild type and mutant (I-*sjdR*/WT) is also shown. The bold italic values indicate significant changes, as assessed by the Student’s *t* test (*P* < 0.05), calculated from the atoms/OD_750_ values.

b Note that the value for atom/cell is shown for orientation, since this value is more error-prone than atoms/OD_750_.

Calcium signaling is essential for heterocyst differentiation and some stress responses in *Anabaena* (56–59). The extracellular calcium concentration strongly influences heterocyst frequency (60, 61) and the expression of heterocyst-specific genes, including the genes coding for the nitrogenase complex (58). Considering the elevated intracellular calcium levels and following the hypothesis of an increased demand for calcium in the I-*sjdR* mutant strain, the impact of an increased calcium supply on the growth of the *sjdR* mutant on YBG11_0_ plates was analyzed. Notably, supplementation with calcium chloride partially complemented the phenotype of the I-*sjdR* mutant in a concentration-dependent manner (Fig. 4D). The mutant strain regained growth on medium without combined nitrogen when calcium concentrations exceeded the standard concentration of 245 μM, and the biomass produced was highest at 5 mM calcium (Fig. 4D). Notably, a calcium-dependent improvement of I-*sjdR* growth was also observed in diazotrophic liquid cultures (see Fig. S2B in the supplemental material). This phenotypic recovery was not observed for the I-*exbB2* and I-*alr1655* control mutants (Fig. 4D). On the other hand, a similar experiment was conducted with elevated concentrations of molybdenum, but those did not restore growth of the mutant under diazotrophic conditions (Fig. 4E).

### The I-*sjdR* mutant exhibits an altered cell morphology and heterocyst pattern.

**FIG 5:**
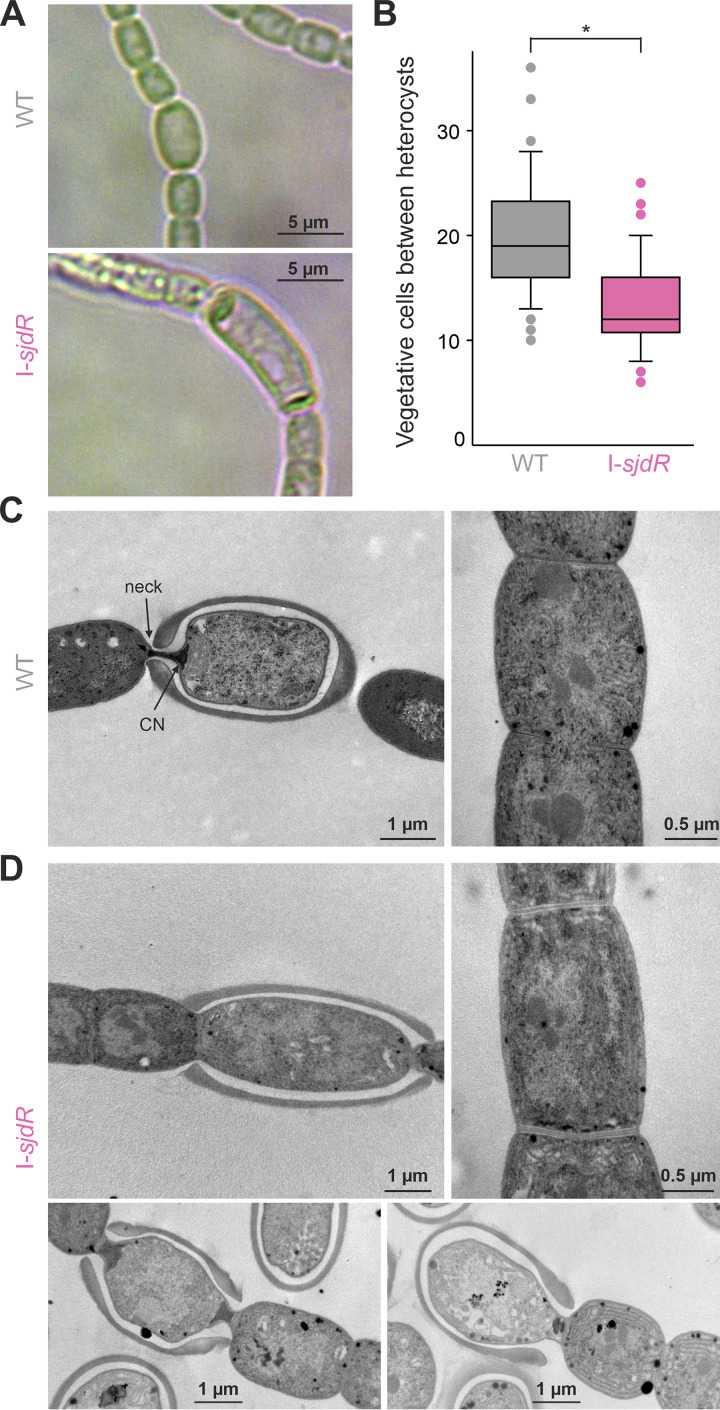
The *sjdR* mutant heterocysts are elongated and have an altered septum structure. (A) Wild-type (WT) and I-*sjdR* filaments were incubated under diazotrophic conditions and inspected by light microscopy. (B) The number of vegetative cells between two heterocysts after growth for 7 days in YBG11_0_ medium was analyzed (*n* = 1,389 for WT cells and *n* = 1,047 for I-*sjdR* cells) in *n* > 70 randomly selected filaments. (C and D) Transmission electron microscopy images from wild-type (C) and I-*sjdR* (D) heterocysts (left and bottom) and vegetative cells (right) are shown. In the image of the wild-type heterocyst, the cyanophycin polymer (CN) and the heterocyst neck are indicated. For I-*sjdR*, multiple heterocysts are shown to illustrate the variability of heterocyst’s ultrastructure.

The loss of nitrogenase activity in the I-*sjdR* mutant strain and the reduced growth in YBG11_0_ medium prompted us to inspect microscopically the I-*sjdR* filaments. Differentiated heterocysts were observed in the I-*sjdR* strain when grown in YBG11_0_ medium (Fig. 5A). Heterocyst frequency, determined after 7 days of growth in nitrogen-depleted medium, was higher in the I-*sjdR* mutant than in the wild type (Fig. 5B). On average, 20 vegetative cells were counted between two heterocysts in the wild type, whereas 13 vegetative cells separated two heterocysts in I-*sjdR* (Fig. 5B). A higher heterocyst frequency is consistent with the impaired nitrogenase activity observed in the I-*sjdR* mutant, which leads to insufficient nitrogen assimilation and triggers the differentiation of an increased number of vegetative cells into heterocysts.

Notably, the morphology of the heterocysts in I-*sjdR* filaments was different from wild-type heterocysts (Fig. 5A). In the I-*sjdR* mutant strain, heterocysts appeared elongated and less ovoid in shape compared to heterocysts of the wild type. Moreover, transparent structures inside the heterocysts were observed in larger numbers in I-*sjdR* heterocysts. In transmission electron microscopy images, the characteristic polar neck and the cyanophycin plug near the poles were visible in wild-type heterocysts (Fig. 5C). Heterocysts of the I-*sjdR* mutant exhibited a certain degree of morphological heterogeneity, but they were characterized by (i) a cyanophycin plug that was barely visible or poorly structured, and (ii) a septum that was expanded, with the contact area to adjacent vegetative cells being larger than in the wild type (Fig. 5D). The morphology of I-*sjdR* vegetative cells was altered compared to the wild type, as the vegetative cells of the mutant were more rectangular (Fig. 5D, right; Table 2). The ratio of the width in the center of the cell to the width of the septum was 1.5 ± 0.1 (*n* = 20) for the wild type and 1.1 ± 0.1 (*n* = 29) for the I-*sjdR* mutant. Considering the strong morphological alterations of the *sjdR* mutant heterocysts compared to vegetative cells, we suggest that the absence of SjdR affects a mechanism or activity that is especially important in heterocysts or for heterocyst formation.

**TABLE 2:** Properties of the septa (Fig. 5), septal disks, and nanopores (Fig. 8) in vegetative cells of the wild-type and I-*sjdR* strains

Characteristic	Value for characteristic^*a*^			
	NO_3_^−^		N_2_	
	WT	I-*sjdR*	WT	I-*sjdR*
Septum width (diam)^*b*^	1.1 ± 0.2 μm (20)	***1.6 ± 0.3 μm*** (29)	ND	ND
Distance between plasma membranes^*b*^	32 ± 8 nm (8)	***58 ± 12 nm*** (15)	ND	ND
Disk diam (DD)	1.2 ± 0.2 μm (12)	***1.6 ± 0.6 μm*** (9)	1.4 ± 0.5 μm (14)	2.3 ± 0.8 μm (9)
Nanopore zone diam (NZD)	0.56 ± 0.08 μm (12)	0.5 ± 0.1 μm (9)	0.7 ± 0.2 μm (14)	0.4 ± 0.3 μm (9)
NZD/DD	0.47 ± 0.1 (12)	***0.31 ± 0.1*** (9)	0.51 ± 0.12 (14)	0.15 ± 0.05 (9)
Nanopore diam	23 ± 5 nm (270)	23 ± 6 nm (129)	24 ± 5 nm (349)	21 ± 4 nm (168)
No. of nanopores per disk	39 ± 9 (12)	***28 ± 9*** (8)	63 ± 31 (14)	53 ± 19 (10)

a Average values and standard deviations are given, and the number of replicates/measurements is indicated in parentheses. The bold italic values of the mutant shown are significantly different from the wild-type (WT) values (Student’s *t* test, *P* < 0.05). ND, not determined; diam, diameter.

b Measurements conducted on TEM micrographs as illustrated in Fig. 5, the other parameters were determined from isolated peptidoglycan sacculi as presented in Fig. 8

### SjdR is required for the formation of heterocyst polar structures.

**FIG 6:**
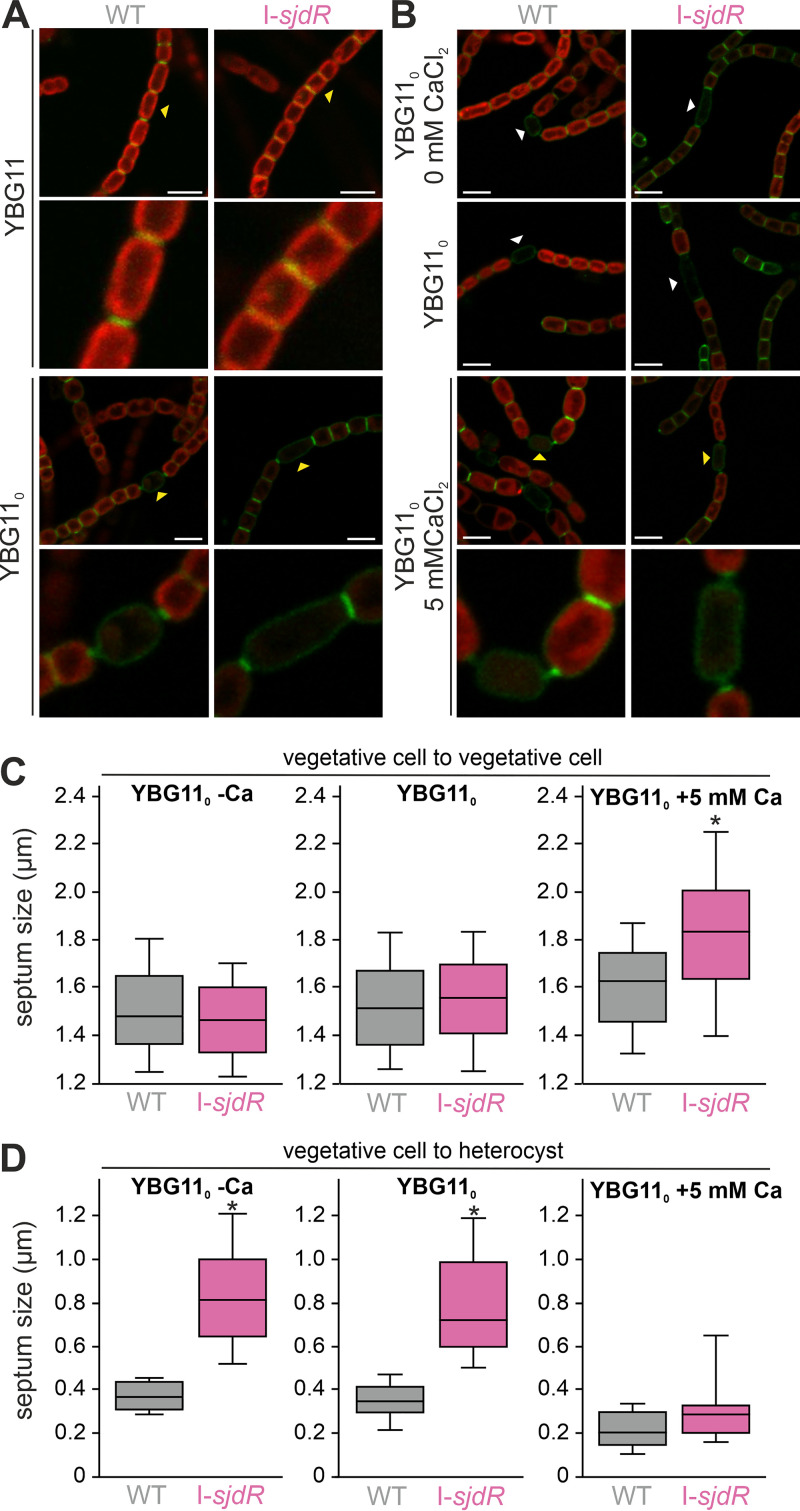
Impaired PG formation during heterocyst differentiation in the I-*sjdR* mutant strain and restoration by an enhanced calcium concentration. (A and B) Wild-type and I-*sjdR* filaments were grown on plates composed of the indicated media and fluorescently labeled with vancomycin-FL for visualizing the peptidoglycan. Merge images of vancomycin-FL fluorescence and autofluorescence are shown. Due to low Chl autofluorescence, in some images of the I-*sjdR* mutant, the intensity was adjusted for better visualization. Representative images are shown. White arrowheads point to heterocysts. Yellow arrowheads point to cells that are fourfold magnified in the panel below. Bars, 5 μm. (C and D) Quantification of the septum sizes between vegetative cells (C) or between vegetative cells and heterocysts (D) from wild-type and I-*sjdR* strains in YBG11_0_ without CaCl_2_ (YGB11_0_ -Ca), YBG11_0_, or YBG11_0_ supplemented with 5 mM CaCl_2_ (YBG11_0_ +5 mM Ca). The measurements were taken from confocal microscope images (representative images shown in panels A and B); >100 septa of vegetative cells were measured per strain and condition, and >18 heterocyst septa were analyzed. The median values are presented in each box, and the error bars show the 95% confidence interval. Statistical analysis was done with ANOVA and Duncan’s multiple range test (independent tests for panels C and D), and the asterisks above the boxes represent the samples that are significantly different from the other sample set (i.e., mutant versus wild type; *P* < 0.001).

Spatial regulation of PG synthesis and distribution is a main determinant of cellular morphology in bacteria (62). Thus, the morphological phenotype of the I-*sjdR* mutant strain might be related to an altered regulation of PG synthesis in the mutant. To test this, PG of the wild-type and mutant strains was fluorescently labeled with vancomycin-FL (63). Cells grown on solid medium were labeled and analyzed by confocal microscopy (Fig. 6). The distribution of the fluorescent dye surrounding a vegetative cell was comparable in the I-*sjdR* mutant and the wild type (Fig. 6A, upper panel). For heterocysts of the wild type, the characteristic polar neck and protuberance of the PG into the adjacent vegetative cell were observed (Fig. 6A, lower panel; yellow arrowheads mark heterocysts that are magnified in the panel below). The PG distribution in heterocyst septa of the I-*sjdR* mutant resembles, with its increased size, the PG architecture between vegetative cells. Therefore, the common PG area at the intercellular septa, also termed septal disk area, between heterocysts and adjacent vegetative cells was larger in the I-*sjdR* mutant than in the wild type. Thus, septal disks appear to be wider along the filament in the mutant than in the wild type, resulting in aberrant septal disks that do not show the characteristic constriction in vegetative cell-heterocyst septa.

As the supplementation of calcium did complement the growth of the I-*sjdR* mutant strain under diazotrophic conditions (Fig. 4), the impact of calcium on PG formation was analyzed. For both the wild type and the mutant strain, the distribution of the septal PG was comparable when grown on YBG11_0_ plates or on YBG11_0_ plates without calcium (Fig. 6B). However, in the presence of 5 mM calcium, the PG distribution in I-*sjdR* heterocyst septa resembled that of the wild type (Fig. 6B). Thus, an enhanced calcium concentration restores the PG morphology in the I-*sjdR* mutant, producing heterocyst septa functional for diazotrophic growth. To quantify this effect, the septum sizes (diameters) were measured for the wild type and I-*sjdR* mutant in the presence and absence of calcium. Interestingly, the analysis of the vegetative cells did not show a difference in septum width between the two strains in YBG11_0_ or YBG11_0_ without calcium (YBG11_0_ -Ca) (Fig. 6C), whereas in YBG11, the I-*sjdR* vegetative cells septa were larger than in the wild type (Table 2); in YBG11_0_ plus 5 mM CaCl_2_, the I-*sjdR* septum diameter was also larger than that from the wild type, with average values of 1.8 ± 0.3 μm and 1.6 ± 0.2 μm, respectively (Fig. 6C). The quantification of the size of the septum between vegetative cells and heterocysts confirmed the presence of enlarged septa in the *sjdR* mutant strain, with on average 0.8 ± 0.3 μm in YBG11_0_ and 0.8 ± 0.2 μm in YBG11_0_ -CaCl_2_ (compared to less than 0.4 μm in the wild type; Fig. 6D). When 5 mM CaCl_2_ was supplemented, the average I-*sjdR* heterocyst septum width decreased to on average 0.3 ± 0.17 μm, similar to the corresponding wild-type value (0.2 ± 0.09 μm [Fig. 6D]). Therefore, calcium supplementation seems to have a differentiated effect on I-*sjdR* septum widths, and the constriction of the heterocyst septum in the presence of 5 mM CaCl_2_ positively impacts the diazotrophic growth capacity of the strain.

### Cell-cell communication is influenced by the disruption of *sjdR*.

**FIG 7:**
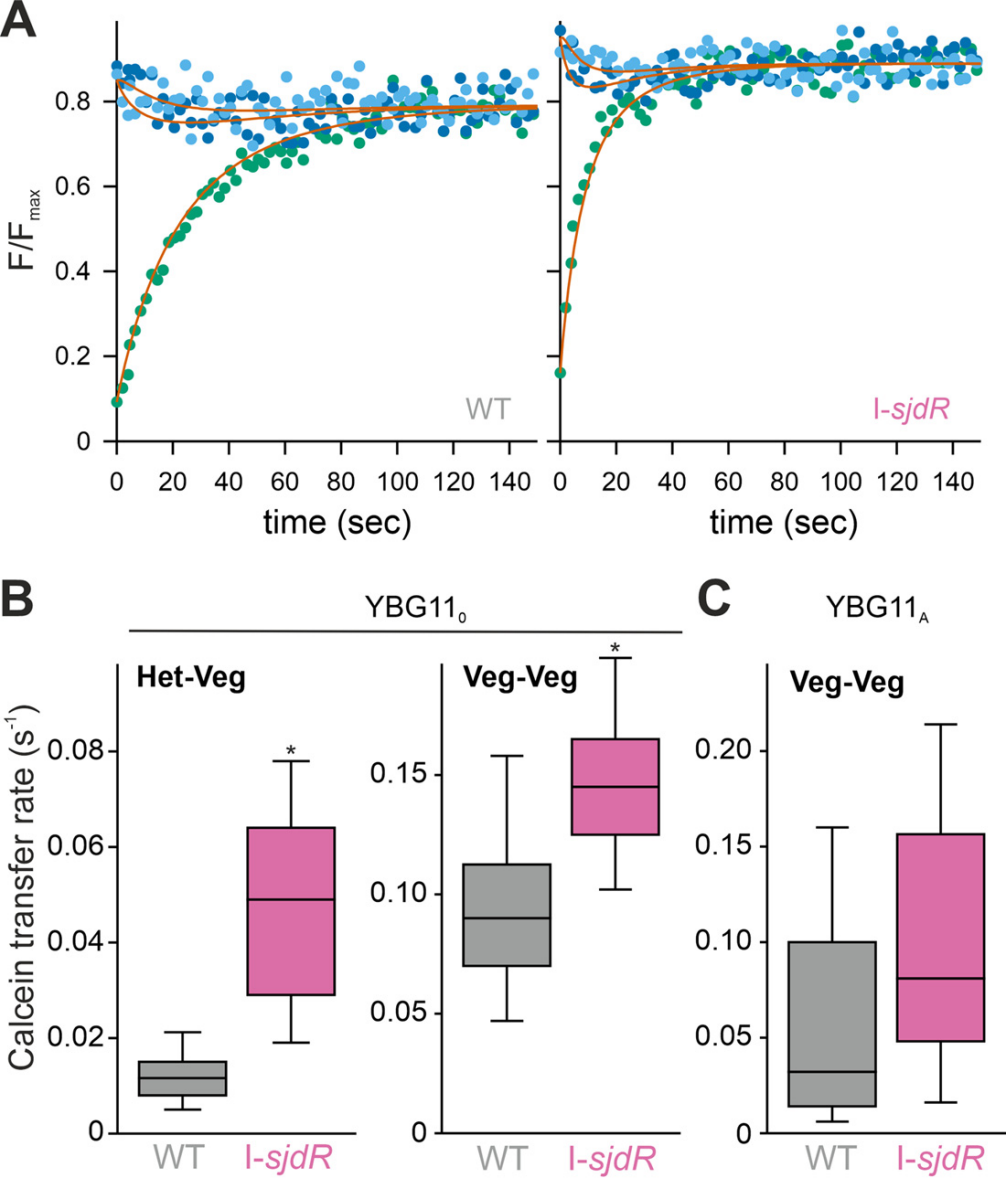
Intercellular calcein transfer rate is increased in the I-*sjdR* mutant strain. (A) The relative fluorescence intensity (F) in single cells was determined after photobleach. Shown is one example for wild-type (WT) (left) and I-*sjdR* (right) strains with the relative fluorescence of the bleached heterocyst visualized in green, the fluorescence of the adjacent vegetative cell in dark blue, and the fluorescence of the second adjacent cell in light blue. The solid line represents the result of the least square fit analysis to the model presented in Materials and Methods for these three cells. (B) The rates of calcein transfer between heterocysts (Het) and vegetative cells (Veg) or between vegetative cells observed for wild type (gray) and I-*sjdR* (pink) strains grown under diazotrophic conditions are shown as box-plots. (C) The calcein transfer rate between vegetative cells in WT (gray) and I-*sjdR* (pink) strains grown in medium containing ammonium is shown. For the box-plots in panels B and C, the central lines indicate the medians, and the error bars represent the 95% confidence intervals.

The septal disk of the I-*sjdR* mutant strain between heterocysts and vegetative cells appears larger than in wild-type *Anabaena* (Fig. 5 and 6), which might have an impact on solute exchange between two adjacent cells. Thus, the rate of solute diffusion was determined using calcein as a fluorescent marker molecule (64). The intercellular diffusion of the marker was recorded after photobleaching of a single cell (Fig. 7A). The fluorescence profiles of the bleached and adjacent cells were determined and analyzed with a 13-cell model (Materials and Methods) providing information for the transfer rate between vegetative cells or between a vegetative cell and a heterocyst.

Under the conditions tested, mean calcein transfer rates between a vegetative cell and a heterocyst were 0.049 s^−1^ and 0.012 s^−1^ for the I-*sjdR* mutant strain and the wild type, respectively (Fig. 7B and Table 3). Therefore, calcein diffusion from vegetative cells into heterocysts was about fourfold faster in the I-*sjdR* mutant compared to the wild type. Similarly, the diffusion rate between vegetative cells of the I-*sjdR* mutant grown in YBG11_0_ medium was higher by a factor of about 1.5 compared to the wild type (Fig. 7B and Table 3). This result was confirmed by analyzing the transfer rate between vegetative cells of some filaments of the two strains grown in YBG11 medium (Table 3, NO_3_^−^) or YBG11 medium supplemented with ammonium (YBG11_A_) to suppress heterocyst formation (Fig. 7C and Table 3, NH_4_^+^). These results clearly show an increased intercellular transfer of calcein in the I-*sjdR* mutant between communicating cells.

**TABLE 3:** Calcein transfer rates for the wild-type and I-*sjdR* strains

Cell type^*a*^	Nitrogen source	Wild type		I-*sjdR*	
		CT rate (s^−1^)^*b*^	*N* ^*c*^	CT rate (s^−1^)^*b*^	*N* ^*c*^
VEG-HET	N_2_	0.012 ± 0.001	68	0.049 ± 0.003	75
VEG-VEG	N_2_	0.094 ± 0.006	53	0.145 ± 0.005	51
VEG-VEG	NO_3_^−^	0.11 ± 0.01	7	0.16 ± 0.02	6
VEG-VEG	NH_4_^+^	0.06 ± 0.02	9	0.11 ± 0.06	8

a VEG, vegetative cells; HET, heterocysts.

b CT rate, calcein transfer rate. The CT rates are shown are as means ± standard errors.

c *N* is the number of experiments.

### The I-*sjdR* mutant strain bears alterations in septal nanopore distribution.

The augmented intercellular molecular transfer in the I-*sjdR* mutant could be related to the larger septa observed in the mutant (Fig. 5 and 6). In *Anabaena*, the velocity of solute exchange depends on the size and number of nanopores and septal junctions (8, 65, 66), which mediate the efficient intercellular exchange of metabolites (67). In order to examine the properties of the nanopores in the I-*sjdR* mutant compared to the wild type, PG sacculi were isolated, and the septal disks were analyzed.

**FIG 8:**
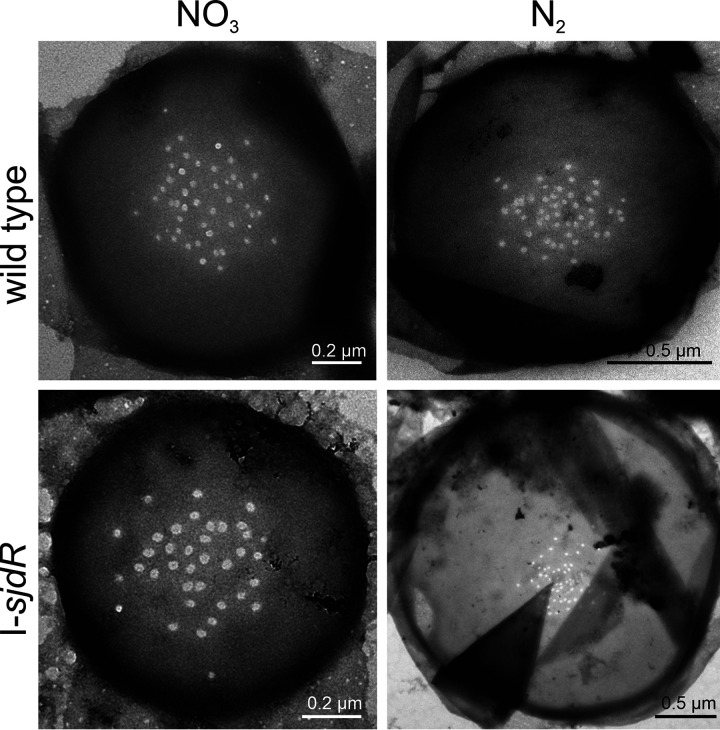
The nanopore distribution in the I-*sjdR* mutant strain. Peptidoglycan sacculi were isolated from BG11-grown filaments or filaments incubated for 48 h in BG11_0_ medium of the wild type and I-*sjdR* mutant. To visualize the septal disks, the sacculi were inspected by transmission electron microscopy as described in Materials and Methods.

The periplasm between adjacent vegetative cells was found to be enlarged in the I-*sjdR* mutant strain as deduced from electron microscopy images (Fig. 5), because both the width of the septum and the distance between the centers of two adjacent plasma membranes were enhanced (Table 2). The same holds true for the septal disks (Fig. 8 and Table 2), in which the effect was most pronounced when the strains were grown in the absence of nitrate. Here, the wild-type septal disks were on average 1.4 ± 0.5 μm in diameter, whereas I-*sjdR* disks had a diameter of 2.3 ± 0.8 μm (Table 2).

Both under nitrate-replete and -deplete conditions, the relative area of the disk in which nanopores were detected was smaller in the *sjdR* mutant. This effect was most drastic in BG11_0_ medium, in which nanopores were found in 3% of the septal disk area in I-*sjdR* but in about 25% of the disk area in the wild type (calculated from the nanopore zone diameter [NZD] and the disk diameter [DD] in Table 2). In both BG11 and BG11_0_ media, the number of nanopores per disk was somewhat lower in the I-*sjdR* mutant strain compared to the wild type. The nanopore diameter was comparable between the two strains in the presence of nitrate, whereas in BG11_0_ medium, the nanopore diameter in the I-*sjdR* mutant was somewhat smaller than in the wild type. Therefore, the increased rate of calcein transfer cannot be explained by an increased number of nanopores in the mutant, but it may result from other factors such as a reduced cyanophycin plug (see Discussion).

In order to investigate the subcellular localization of SjdR, a strain expressing SjdR-GFP was created (Fig. S3). Due to the low expression levels of *sjdR*, the green fluorescent protein (GFP) fluorescence signal was weak under the confocal microscope. A quantification of the fluorescence signal in the septum and the sidewall region showed that GFP was not significantly enriched in the septum (Fig. S3), and SjdR-GFP rather seems to be evenly distributed along the cell envelope. This strengthens the hypothesis that SjdR plays a general role in PG structuring.

10.1128/mBio.00483-21.3 FIG S3 Construction and analysis of the *sjdR-gfp* strain. (A) Illustration of homologous recombination of plasmid pCSV3-sjdR-sf-gfp into the *Anabaena* genome. The genotype of the *sjdR-gfp* mutant strain is indicated at the bottom; the arrowheads indicate annealing sites of the oligonucleotides used for screening. (B) PCR on DNA of the *sjdR-gfp* mutant (Mut) and wild type (WT), an oligonucleotide pair indicating the plasmid insertion (A) or oligonucleotides annealing outside of the homologous region indicating the presence of the wild-type *sjdR* gene (B) were used. The asterisk marks an unspecific PCR product. (C) Schematic illustration of the regions where the GFP fluorescence was measured in the wild type and the *sjdR-gfp* strain. (D) Examples of fluorescence images for WT and *sjdR-gfp* strain, a merge of autofluorescence (red) and GFP (green) channels is shown. The reference bar indicates 5 μm. (E) GFP fluorescence was quantified at regions 1 and 2 as depicted in panel C, and the wild-type values were subtracted from the fluorescence in the mutant. A minimum of 120 measurements at the septum or the sidewall were taken from different filaments of each strain. The error bars indicate the standard deviations. Download FIG S3, TIF file, 2.3 MB. Copyright © 2021 Schätzle et al. 2021 Schätzle et al. https://creativecommons.org/licenses/by/4.0/ This content is distributed under the terms of the Creative Commons Attribution 4.0 International license.

## DISCUSSION

In *Anabaena*, four proteins have been identified as putative TonB-like proteins (44). However, the previously assigned TonB1 (here denoted SjdR) is exceptionally short, and considering the distance between PM and OM (2), this protein would be unable to interact with OM proteins. In addition, SjdR does not contain a TonB-like periplasmic domain, whereas a similarity to the ZipA divisome protein and GltJ/AgmX superfamiliy proteins is predicted instead (Fig. 1). Whether SjdR contributes to gliding motility in *Anabaena* was not analyzed in this study, but it should be noted that *Anabaena* is described as nonmotile (68).

The periplasmic domain of SjdR is rich in hydroxylated amino acids (serine, threonine) and prolines. Notably these amino acids are enriched as well in the SepJ linker domain and in SepI, both of which are septal proteins in *Anabaena* (9, 18). The absence of SjdR in a plasmid insertion *sjdR* mutant of *Anabaena* does not affect the siderophore transport rate, whereas a *tonB3* mutant generated by the same strategy is drastically impaired in siderophore-dependent iron transport (Fig. 2). This is consistent with the idea that TonB3 is central for energizing the ferric siderophore uptake as previously suggested (44). The involvement of SjdR in TonB-dependent transport was rejected based on the analyzed uptake of schizokinen which is produced by *Anabaena* sp., but a participation in the transport of other substrates cannot be excluded. However, considering its structure and length, we suggest that SjdR takes over a function distinct from TonB-dependent transport in *Anabaena*, perhaps a function as an interaction platform for periplasmic proteins involved in PG maintenance as will be discussed below.

The growth of the *sjdR* mutant (I-*sjdR*) is severely delayed in the absence of nitrate (Fig. 2), although nitrogen step-down does not induce *sjdR* transcription (Fig. 3). This argues against a direct involvement of SjdR in heterocyst differentiation. Despite a substantial expression of nitrogenase-encoding genes (tested with *nifH*), nitrogenase activity is absent under oxic conditions but detectable (albeit at low levels) under anoxic conditions (Fig. 3). This suggests that the phenotype of the mutant is related to a breakdown of the microoxic environment in the heterocyst rather than to the absence of nitrogenase production. Heterocysts of the *sjdR* mutant are morphologically altered, since they are elongated and less ovoid compared to those of the wild type (Fig. 5). Further, the vegetative cell-heterocyst connection shows an abnormal architecture in which a wide heterocyst neck is observed and the cyanophycin plug is reduced or missing (Fig. 5). This could result, at least in part, from the compromised nitrogen fixation capacity of the mutant strain. This morphological phenotype is comparable to those of the *hglK* (67) and *conR* (69, 70) mutants, as well as a strain in which the *sepJ* gene is overexpressed (65).

The diffusion of the fluorescent marker calcein between cells is faster in the *sjdR* mutant than in the wild type, which is most pronounced for the transfer between vegetative cells and heterocysts (Fig. 7), again resembling the phenotype of the *hglK* mutant (71). The transfer of solutes between cells first depends on the number and size of nanopores in the septal PG mesh that connect the cytoplasm of adjacent cells in the filament (8, 66). Notably, the number of nanopores between vegetative cells is somewhat lower in the *sjdR* mutant than in the wild type; thus, the faster transfer in the mutant must be grounded on other factors. In addition to nanopore architecture, also other factors influence molecular transfer in *Anabaena*. For example, the inactivation of *glsP* (encoding a glucoside ABC transporter permease subunit) or of *hepP* (encoding a major facilitator superfamily [MFS] glucoside transporter) decreases the calcein transfer rate, but not the number of nanopores (72). Moreover, septal junctions are gated, since different stress conditions can reversibly reduce the diffusion activity (73). Hence, we hypothesize that SjdR affects the proteinaceous components of the septal junctions in a way such that calcein transfer is influenced as observed in the *sjdR* mutant. In addition, the reduced cyanophycin plug in the mutant heterocysts might contribute to the increased rate of calcein diffusion (64). An increased molecular influx into heterocysts could in turn contribute to inactivation of nitrogenase by oxygen entering through the wide heterocyst poles in the mutant (6, 74). Whether there is a direct interaction between SjdR and septal proteins remains to be elucidated. However, since an enrichment of SjdR-GFP was not observed near the septum, SjdR function seems to not be limited to the septum area.

The septal PG arrangement at the poles of the heterocyst in the *sjdR* mutant resembles the one between vegetative cells (Fig. 6). This suggests an important role of SjdR in the formation of the vegetative cell-heterocyst septa. Nonetheless, the altered phenotype of the *sjdR* mutant is not restricted to heterocysts, as in YBG11 medium also, the septal disk area between vegetative cells is enlarged compared to the wild type (Fig. 5 and Table 2). Additionally, the zone occupied by the nanopores in the septal disks of vegetative cells is significantly smaller in the *sjdR* mutant than in the wild type, which represents a novel morphological phenotype (unfortunately, no data are available specifically for the heterocyst-vegetative cell septa). These results imply a relation of SjdR with PG formation or remodeling in *Anabaena*. Spatial PG synthesis is a major determinant of cell shape in bacteria (62). We assume that the differential activity of PG metabolism and plasticity in the two cell types (vegetative cells and heterocysts) is the reason for the morphological *sjdR* mutant phenotype, which is mostly visible in heterocysts. SjdR deletion consequently seems to impact mechanisms that are especially active or required for normal heterocyst formation. In *Anabaena*, PG synthesis is stronger during heterocyst differentiation than in vegetative cells and proceeds along the whole cell, whereas in vegetative cells, it is mainly limited to the division site (75). A deregulation of PG arrangement due to lack of SjdR function can explain the alterations in heterocyst morphology and the alterations in the vegetative cell septa, whereas we do not know whether vegetative cell lateral walls are somehow affected. The proper regulation of PG synthesis and elasticity is essential for heterocyst differentiation (76, 77). On the one hand, heterocyst-specific polysaccharides and lipids need to pass through the cell wall (78), and the PG of heterocysts is thicker than that of vegetative cells (75). Thus, a relation of SjdR to PG organization would be consistent with the Fox^−^ phenotype of the mutant (Fig. 3).

An increase of calcium concentration was observed in the *sjdR* mutant compared to the wild type (Table 1). The growth of the mutant under diazotrophic conditions was largely restored by calcium supplementation (Fig. 4). Since calcium stabilizes the PG and regulates the elongasome activity in some bacteria (79, 80), the *sjdR* mutant might accumulate calcium to compensate for a defect in PG metabolism, with calcium supplementation further enhancing this effect. This is consistent with the reported observations that extracellular calcium has a strong impact on nitrogen metabolism in *Anabaena* (58, 61, 81) and that elevated calcium levels accelerate heterocyst differentiation (81) or enhance the performance of nitrogenase (82). Notably, in *Anabaena*, the intracellular calcium level is about 1 order of magnitude higher in heterocysts than vegetative cells (56, 57).

Proteins with similarity to *Anabaena* SjdR were found to be conserved among cyanobacteria, except for *Gloeobacteria* and *Spirulinales.* As is the case for elangasome- and divisome-related proteins, which define cellular morphology and at the same time are conserved in unicellular and filamentous strains, it is conceivable that the SjdR functionality adapted over time in different cyanobacteria (83, 84).

In summary, the highly conserved SjdR protein (Fig. 1) has a structural role in the maintenance of cell wall morphology in *Anabaena*. SjdR knockout induces an altered PG distribution, which becomes evident in the aberrant morphology of septal PG disks and, more generally, in cell shape alterations and impairment of diazotrophic growth. The latter is likely the consequence of an increased oxic environment in the heterocysts that results from the increased septum size, permitting an elevated influx of solutes, including oxygen.

## MATERIALS AND METHODS

### *In silico*/bioinformatics analyses.

Cyanobacterial sequences were collected by BLAST search (NCBI with the following settings: BLOSUM62; gap costs: existence, 11, extension, 1; 500 sequences) using the SjdR and TonB3 protein sequences from *Anabaena* sp. strain PCC 7120 as bait (85, 86). All sequences identified were filtered for redundancy using cd-hit (87) with a setting of 90% sequence identity. The distribution of sequences in different species is shown according to the taxonomy nomenclature deposited in NCBI (88). Domains of SjdR were extracted from NCBI (89). The transmembrane domain was predicted by TMHMM (90).

### *Anabaena* culture conditions.

*Anabaena* wild type and mutants were stored on BG11 medium agar plates (68) containing 1% (wt/vol) Bacto agar (BD Biosciences). Liquid and solid media for mutants were supplemented to 5 μg ml^−1^ of each spectinomycin dihydrochloride pentahydrate (Duchefa Biochemie) and streptomycin sulfate (Roth). For liquid cultures, BG11 or buffered YBG11 medium was utilized (91). No precipitation of compounds is observed in YBG11, which in experiments that address questions about metal availability is an important condition. For YBG11_0_ (nitrate-free YBG11), CoCl_2_ was used instead of CoNO_3_.

Cultures were grown with constant shaking (90 to 100 rpm) under permanent illumination at 28°C and 70 μmol photons m^−2^ s^−1^ (Frankfurt) or at 30°C and about 25 μmol photons m^−2^ s^−1^ (Seville; for nitrogenase activity determination and septal disk isolation). Bubbling cultures were enriched with CO_2_ (1%) and 10 mM NaHCO_3_ was used for pH stabilization of the medium. Growth analysis was done on agar plates spotted with 5 μl of cell suspensions previously adjusted to an optical density at 750 nm (OD_750_) of 1 or 0.1 without antibiotics.

### DNA extraction and molecular cloning.

Genomic DNA from *Anabaena* was isolated as described previously (92) with the following modifications: sodium dodecyl sulfate (SDS) was not added to the samples, and the phenol extraction was done once, followed by two washing steps with 400 μl chloroform.

### Generation of *Anabaena* mutants.

Single recombinant mutants were created as described previously (93–96). In the case of AFS-I-*sjdR* (I-*sjdR*), an *sjdR*-internal fragment of 532 bp was amplified by PCR (oligonucleotides [see Table S1 in the supplemental material]), and EcoRI and EcoRV restriction sites were inserted at the 5′ and 3′ ends, respectively. The fragment was inserted into EcoRI/EcoRV-digested pCSEL24 that contains a CS.3 cassette (95). The plasmids are listed in Table S3. In the case of AFS-I-*tonB2* (I-*tonB2*) and AFS-I-*tonB4* (I-*tonB4*), fragments of 467 bp and 584 bp, respectively, were amplified, and BglII sites were introduced at both ends. For cloning AFS-I-*exbB2* (I-*exbB2*), an internal fragment of 408 bp was amplified, and BamHI sites were inserted at both ends. All fragments were ligated into BamHI-digested pCSV3. pCSV3 is a modified version of pRL500 (97) bearing the CS.3 resistance cassette (98). The plasmids (Table S3) were transferred to wild-type *Anabaena* by conjugation as previously described (94) utilizing Escherichia coli strains HB101 and ED8654. Segregation of mutant strains was tested utilizing an oligonucleotide specific for the plasmid in combination with oligonucleotides annealing outside the internal fragment (Fig. 1; see also Fig. S1 in the supplemental material). All the mutant strains are listed in Table S2. The AFS-I-*all1636* (I-*all1636*) and AFS-I-*alr1655* (I-*alr1655*) mutants were a gift from L. Fresenborg.

10.1128/mBio.00483-21.1 FIG S1 Genotypes of mutants utilized in this study. Genomic DNA from the wild type (WT) and I-*tonB2* (A), I-*tonB3* (B), I-*tonB4* (C), and I-*exbB2* (D) mutants was used as the PCR template. Oligonucleotides used for screening are listed in Table S1. The *tonB3* mutant was already mentioned in previous studies (44). For each mutant, an oligonucleotide combination specific for the gene of interest (lanes marked with B) or a combination where one oligonucleotide anneals in the plasmid used for insertion and the other oligonucleotide anneals in the gene (lanes marked with A) was used (imaged in Fig. 1). The gene-specific fragment in lanes B is expected in the wild-type strain and also in nonsegregated mutants, whereas fragment in lanes A is expected only in the mutant strains. I-*tonB2* and I-*exbB2* mutants are segregated, whereas I-*tonB3* and I-*tonB4* mutants are not segregated. The asterisk in panel C indicates an unspecific PCR product. Download FIG S1, TIF file, 1.3 MB. Copyright © 2021 Schätzle et al. 2021 Schätzle et al. https://creativecommons.org/licenses/by/4.0/ This content is distributed under the terms of the Creative Commons Attribution 4.0 International license.

10.1128/mBio.00483-21.5 TABLE S2 *Anabaena* sp. strains used in this study. Download Table S2, DOCX file, 0.01 MB. Copyright © 2021 Schätzle et al. 2021 Schätzle et al. https://creativecommons.org/licenses/by/4.0/ This content is distributed under the terms of the Creative Commons Attribution 4.0 International license.

10.1128/mBio.00483-21.6 TABLE S3 Plasmids used in this study. Download Table S3, DOCX file, 0.01 MB. Copyright © 2021 Schätzle et al. 2021 Schätzle et al. https://creativecommons.org/licenses/by/4.0/ This content is distributed under the terms of the Creative Commons Attribution 4.0 International license.

To analyze the subcellular localization of SjdR, the superfolder GFP (*sf-gfp*) gene was fused to the 3′ end of the *sjdR* open reading frame, and a sequence encoding a tetraglycine linker was inserted between *sjdR* and *sf-gfp*. Molecular cloning was performed as described previously (71). In brief, the *sf-gfp* sequence was amplified without the start codon (see oligonucleotides in Table S1), an EcoRV site as well as the tetraglycine-encoding sequence was added at the 5′ end, and a KpnI site at the 3′ end. The *gfp-mut2* sequence was excised from plasmid pCSEL21 (95) (Table S3) with KpnI/EcoRV, and *sf-gfp* was inserted instead, yielding pCSEL21-sf-gfp. The *sjdR* sequence was amplified by PCR utilizing *Anabaena* genomic DNA as the template; the stop codon was not included. ClaI and EcoRV sites were inserted at the 5′ and 3′ ends, respectively, and the sequence was inserted into pCSEL21-sf-gfp after restriction digest with ClaI/EcoRV. After the insertion of the *sjdR-sf-gfp* fusion into pCSEl21 was verified by sequencing, *sjdR-sf-gfp* was excised with EcoRI and ligated into EcoRI-digested pCSV3. The resulting plasmid pCSV3-sjdR-sf-gfp (Table S2) was transferred to *Anabaena* (described above), and the genotype of the *sjdR-gfp* strain (Table S2) was analyzed by PCR.

### Short-term siderophore transport measurements and chlorophyll measurements.

Ferric schizokinen uptake experiments were conducted as described earlier (99, 100). In brief, ^55^FeCl_3_ (PerkinElmer) was complexed to a threefold excess of iron-free schizokinen (EMC Microcollections). A final concentration of 15 nM ^55^Fe-schizkonen was utilized as the substrate. For the uptake experiments, the OD_750_ of the culture was adjusted to 0.05. The last samples were taken ∼3.5 h after the addition of substrate. *Anabaena* strains were grown in iron-depleted medium prior to the uptake experiments. The cellular concentration of chlorophyll *a* (Chl) is an indicator for the degree of iron deficiency in *Anabaena*, since it constantly decreases with ongoing starvation (43). Chl content was divided by the OD_750_ (Table S4), which allowed a comparison of the starvation levels of different cultures. Only strains that exhibited a comparable Chl at OD_750_ of 1 were utilized. Chl concentration was determined in methanolic extracts as previously described (101) and was calculated with the following formula: Chl (in milligrams per milliliter) = *A*_665_/74.5.

### Calcein staining and fluorescence recovery after photobleaching.

YBG11_0_ cultures used for calcein staining were grown for 3 days in YBG11_0_ in shaken flasks. Cultures grown in YBG11_A_ or YBG11 medium were grown in bubbling cultures for 3 days. Staining of *Anabaena* cells with calcein-AM (Invitrogen) was done according to reference 64. For fluorescence microscopy, 50 to 150 μl of cell suspension was spotted onto YBG11, YBG11_A_, or YBG11_0_ agar plates. After drying of the liquid, a small portion of agar was excised and reversely placed onto a coverslip that was utilized as microscope slide. Fluorescence recovery after photobleaching (FRAP) was measured at 23°C with a Zeiss LSM 780 using a 40× oil immersion objective. Excitation with an argon laser at 488 nm was set at 100% for bleaching, and the laser power was reduced to 20% for further recording. Emission was measured at 500 to 540 nm, and the pinhole diameter was set at 180.5 μm. Two or three scans were recorded prior to bleaching, and recovery was recorded for at least 60 s. Only communicating cells were analyzed, since noncommunicating cells occurred in low frequency both in the wild type and the mutants under the conditions used in this study. The data for calcein transfer between vegetative cells were fitted using a reaction chain of exchange between 13 cells according to the following reaction between two cells: [*C*_1_ ← *k_V_* → *C*_2_]_13_ with *C*_1_ being the fluorescence equivalent to the concentration of calcein in cell 1, *C*_2_ being the fluorescence equivalent to the concentration of calcein in cell 2, and *k_V_* being the rate for the forward and backward transfer of calcein for a chain of 13 vegetative cells with the bleached cell as the center. The values for the bleached and neighboring cells were used as input parameters.

The data for transfer into heterocysts were fitted using a reaction scheme between six vegetative cells on each side of the heterocyst as well as between the heterocyst and the neighboring vegetative cells:

$${\left[C_{1}\leftarrow k_{V}\to C_{2}\right]}_{6}\leftarrow k_{\text{VH}}\to C_{H}\leftarrow \;k_{\text{VH}}\to \;{\left[C_{1\prime }\leftarrow \;k_{V}\to C_{2\prime }\right]}_{6}$$

with *C*_1_/*C*_1′_ being the fluorescence equivalent to the concentration of calcein in vegetative cell 1 or cell 1′, *C*_2_/*C*_2′_ being the fluorescence equivalent to the concentration of calcein in vegetative cell 2 or cell 2′, and *k_V_* being the rate for the forward and backward transfer of calcein for a chain of six vegetative cells with the bleached heterocyst as center. *C_H_* is the fluorescence equivalent to the concentration of calcein in the heterocyst, and *k*_VH_ is the rate for the forward and backward transfer of calcein between heterocyst and vegetative cell. A similar rate for forward and backward exchange is assumed, as calcein is a nonnative substrate. In the case of a filament with a terminal heterocyst, the reaction was adapted to:

$${\left[C_{1}\leftarrow k_{V}\to C_{2}\right]}_{6}\leftarrow k_{\text{VH}}\to C_{H}$$

### Vancomycin-FL staining and microscopy.

For confocal and electron microscopy, the strains were grown for 7 days in the indicated medium, since a suitable cell density was required for performing the experiments. Cells were stained with BODIPY FL vancomycin (Invitrogen) as described previously (63). Following two washing steps, the cells were spotted onto YBG11 or YBG11_0_ agar plates and incubated for 90 min at 28°C in darkness. A piece of agar was excised afterwards, reversely placed onto a coverslip, and imaged with a Zeiss LSM 780 using a 63× or 40× oil immersion objective. The pinhole diameter was set at 69.4 μm, excitation was done with an argon laser at 488 nm, and fluorescence was tracked between 500 and 550 nm. Chl autofluorescence was monitored in the range of 630 to 700 nm. Light microscope images were taken with Olympus CKX41.

The quantitative analysis of the GFP fluorescence of the *sjdR-gfp* strain was performed as previously described with modifications (72). Since the GFP fluorescence was low in these experiments, the wild-type and *sjdR-gfp* strains were cultured in iron-free medium prior to the analysis, because we observed an increase of the signal compared to normal conditions. The parameter settings are described above. Quantification of the fluorescence was done as follows: the integrated density in squares of 1 × 1 μm was measured with ImageJ, and a minimum of 120 measurements were taken for each septum and sidewall regions from different filaments. The background value was measured in each picture and subtracted from the corresponding septum or sidewall values. The average wild-type value was subtracted from the average value of the *sjdR-gfp* strain.

Samples for transmission electron microscopy were prepared as reported previously (67) with modifications. In brief, cells were harvested by centrifugation (1,000 × *g*, 5 min), transferred into microcentrifuge tubes, and pelleted again. Cells were resuspended in 2 ml fixation buffer (0.08 M sodium cacodylate [pH 7.3], 2% glutaraldehyde) and incubated for at least 2 h at 4°C. Afterwards, cells were washed twice by centrifugation (1,000 × *g*, 5 min) with wash buffer (0.08 M sodium cacodylate [pH 7.3], 10% [wt/vol] sucrose). Fixation was done in 1% OsO_4_ for 1 h, and the dehydration was performed using ethanol gradient series up to 100% ethanol. Then samples were incubated in propylene oxide and infiltrated overnight with a mixture of propylene oxide and araldite (1:1) and embedded in araldite the next day. Ultrathin sections of about 50 nm were stained with uranyl acetate and lead citrate. A Zeiss EM 900 transmission electron microscope was used for visualization.

### RNA, cDNA synthesis, and qRT-PCR.

RNA extraction was performed as described using TRIzol from Thermo Fisher Scientific (102). The absence of DNA in the RNA sample was tested by PCR using oligonucleotides specific for the *rnpB* gene (Table S1). cDNA synthesis was done with Revert Aid Transcriptase (Thermo Fisher Scientific) according to the manufacturer’s instructions using 1.5 μg of total RNA as the template.

qRT-PCR was performed utilizing StepOnePlus Cycler (Thermo Fisher Scientific). Gene-specific oligonucleotides (Table S1) were added to a concentration of 0.3 μM. Template cDNA was diluted at least 1:3, and PowerUp SYBR green Master Mix (Applied Biosystems) was utilized according to the manufacturer’s protocol. Cycling conditions were set to 2 min at 50°C and 2 min at 95°C for 2 min initially, followed by 40 cycles with 1 cycle consisting of 15 s at 95°C, 30 s at 60°C, and 30 s at 72°C. As a control housekeeping gene, *rnpB* was used.

### Nitrogenase activity.

Nitrogenase activity was determined by the acetylene reduction assay (103). Filaments were grown in BG11 medium, harvested by centrifugation, washed with BG11_0_, inoculated at 1 μg Chl ml^−1^ in 25 ml of BG11_0_ (without antibiotics), and incubated under culture conditions for 48 h. Assays were performed with 2 ml of cell suspension (6 μg ml^−1^) in sealed flasks (total volume, 14 to 17 ml) under both oxic and anoxic conditions. The reaction was started by injecting a saturating amount of acetylene (1 ml). One-milliliter samples for determination of ethylene by gas chromatography were taken for up to 2 h. For anoxic conditions, the cells were supplemented with 10 μM 3-(3,4-dichlorophenyl)-1,1-dimethylurea (DCMU), bubbled with argon for 4 min, and incubated for 60 min before starting the reaction.

### Inductively coupled plasma mass spectrometry.

Glassware was incubated in 4% HNO_3_ overnight. Strains were grown for 7 days in YBG11, harvested by centrifugation (3,000 × *g*, 10 min), and washed in 20 mM 2-(*N*-morpholino)ethanesulfonic acid (pH 5) and 10 mM ethylenediaminetetraacetic acid (104). After the second washing step, the pellet was resuspended in 5 ml double-distilled water (ddH_2_O). For normalization, cells were counted using a Helber bacterial counting chamber (Hawksley), and OD_750_ was measured. Subsequent experiments were conducted in a metal clean laboratory. A volume of 1 ml of each sample was incubated at 120°C in 7 M HNO_3_ overnight until dryness. Before measurement, the samples were dissolved in 5% HNO_3_. As controls, samples of ddH_2_O and culture medium were analyzed.

### Peptidoglycan isolation and septal disk visualization.

Filaments were grown in liquid BG11 medium (supplemented with antibiotics as required) to about 3 to 4 μg Chl ml^−1^, harvested, washed with BG11_0_ medium, inoculated at the same cell density, and incubated in BG11_0_ medium (without antibiotics) for 48 h. Cultures were harvested by centrifugation and fragmented in a sonication bath, and the sacculi were isolated by boiling in the presence of SDS and treatment with α-chymotrypsin (from bovine pancreas; Sigma) following the protocol described (105). The purified sacculi were deposited on copper grids coated by Formvar/carbon film (Aname) and stained with 1% (wt/vol) uranyl acetate for 15 min. All the samples were examined with a ZEISS LIBRA 120 PLUS electron microscope at 120 kV.
