# Supplementary material for: A TonB-Like Protein, SjdR, Is Involved in the Structural Definition of the Intercellular Septa in the Heterocyst-Forming Cyanobacterium Anabaena
Source: mBio. 2021 Jun 8;12(3):e00483-21. doi: 10.1128/mBio.00483-21 (PMC8262864; doi:10.1128/mBio.00483-21)
Supplement: TABLE S5 [file mbio.00483-21-st005.docx]

**Table S5: Cyanobacteria with SjdR-like sequences**

| Order | Family | Genus | Species/Strain |  |
| --- | --- | --- | --- | --- |
| Nostocales | Nostocaceae | Nostoc | unclassified Nostoc | Nostoc sp. ATCC 43529 |
| Nostocales | Nostocaceae | Nostoc | unclassified Nostoc | Nostoc sp. ATCC 53789 |
| Nostocales | Nostocaceae | Nostoc | unclassified Nostoc | Nostoc sp. CENA543 |
| Nostocales | Nostocaceae | Nostoc | unclassified Nostoc | Nostoc sp. HK-01 |
| Nostocales | Nostocaceae | Nostoc | unclassified Nostoc | Nostoc sp. KVJ20 |
| Nostocales | Nostocaceae | Nostoc | unclassified Nostoc | Nostoc sp. 'Lobaria pulmonaria (5183) cyanobiont' |
| Nostocales | Nostocaceae | Nostoc | unclassified Nostoc | Nostoc sp. MBR 210 |
| Nostocales | Nostocaceae | Nostoc | unclassified Nostoc | Nostoc sp. NIES-4103 |
| Nostocales | Nostocaceae | Nostoc | unclassified Nostoc | Nostoc sp. PA-18-2419 |
| Nostocales | Nostocaceae | Nostoc | unclassified Nostoc | Nostoc sp. PCC 7107 |
| Nostocales | Nostocaceae | Nostoc | unclassified Nostoc | Nostoc sp. PCC 7120 |
| Nostocales | Nostocaceae | Nostoc | unclassified Nostoc | Nostoc sp. PCC 7524 |
| Nostocales | Nostocaceae | Nostoc | unclassified Nostoc | Nostoc sp. 'Peltigera membranacea cyanobiont' 210A |
| Nostocales | Nostocaceae | Nostoc | unclassified Nostoc | Nostoc sp. 'Peltigera membranacea cyanobiont' 232 |
| Nostocales | Nostocaceae | Nostoc | unclassified Nostoc | Nostoc sp. 'Peltigera malacea cyanobiont' DB3992 |
| Nostocales | Nostocaceae | Nostoc | unclassified Nostoc | Nostoc sp. 'Peltigera membranacea cyanobiont' N6 |
| Nostocales | Nostocaceae | Nostoc | unclassified Nostoc | Nostoc sp. RF31YmG |
| Nostocales | Nostocaceae | Nostoc | unclassified Nostoc | Nostoc sp. T09 |
| Nostocales | Nostocaceae | Nostoc | unclassified Nostoc | Nostoc sp. TCL240-02 |
| Nostocales | Nostocaceae | Nostoc | unclassified Nostoc | Nostoc sp. UIC 10630 |
| Nostocales | Nostocaceae | Nostoc | Nostoc calcicola |  |
| Nostocales | Nostocaceae | Nostoc | Nostoc carneum |  |
| Nostocales | Nostocaceae | Nostoc | Nostoc commune |  |
| Nostocales | Nostocaceae | Nostoc | Nostoc cycadae |  |
| Nostocales | Nostocaceae | Nostoc | Nostoc flagelliforme |  |
| Nostocales | Nostocaceae | Nostoc | Nostoc linckia |  |
| Nostocales | Nostocaceae | Nostoc | Nostoc minutum NIES-26 |  |
| Nostocales | Nostocaceae | Nostoc | Nostoc piscinale |  |
| Nostocales | Nostocaceae | Nostoc | Nostoc punctiforme NIES-2108 |  |
| Nostocales | Nostocaceae | Nostoc | Nostoc punctiforme |  |
| Nostocales | Nostocaceae | Nostoc | Nostoc sphaeroides |  |
| Nostocales | Nostocaceae | Nostoc | Nostoc sphaeroides CCNUC1 |  |
| Nostocales | Nostocaceae | Anabaena | unclassified Anabaena | Anabaena sp. |
| Nostocales | Nostocaceae | Anabaena | unclassified Anabaena | Anabaena sp. UHCC 0204 |
| Nostocales | Nostocaceae | Anabaena | unclassified Anabaena | Anabaena sp. UHCC 0253 |
| Nostocales | Nostocaceae | Cylindrospermum | Cylindrospermum stagnale |  |
| Nostocales | Nostocaceae | Cylindrospermum | unclassified Cylindrospermum | Cylindrospermum sp. NIES-4074 |
| Nostocales | Aphanizomenonaceae | Anabaenopsis | Anabaenopsis circularis |  |
| Nostocales | Aphanizomenonaceae | Nodularia | unclassified Nodularia | Nodularia sp. |
| Nostocales | Calotrichaceae | Calothrix, Calothrix brevissima |  |  |
| Nostocales | Calotrichaceae | Calothrix, Calothrix desertica |  |  |
| Nostocales | Calotrichaceae | Calothrix | unclassified Calothrix | Calothrix sp. 336/3 |
| Nostocales | Calotrichaceae | Calothrix | unclassified Calothrix | Calothrix sp. HK-06 |
| Nostocales | Calotrichaceae | Calothrix | unclassified Calothrix | Calothrix sp. NIES-2100 |
| Nostocales | Calotrichaceae | Calothrix | unclassified Calothrix | Calothrix sp. NIES-2098 |
| Nostocales | Calotrichaceae | Calothrix | unclassified Calothrix | Calothrix sp. NIES-4101 |
| Nostocales | Calotrichaceae | Calothrix | unclassified Calothrix | Calothrix sp. PCC 7103 |
| Nostocales | Chlorogloeopsidaceae | Chlorogloeopsis | Chlorogloeopsis fritschii |  |
| Nostocales | Hapalosiphonaceae | Fischerella | unclassified Fischerella | Fischerella sp. PCC 9431 |
| Nostocales | Hapalosiphonaceae | Fischerella | Fischerella muscicola |  |
| Nostocales | Hapalosiphonaceae | Fischerella | Fischerella thermalis |  |
| Nostocales | Hapalosiphonaceae | Hapalosiphon | unclassified Hapalosiphon | Hapalosiphon sp. MRB220 |
| Nostocales | Scytonemataceae | Scytonema | unclassified Scytonema | Scytonema sp. HK-05 |
| Nostocales | Scytonemataceae | Scytonema | unclassified Scytonema | Scytonema sp. UIC 10036 |
| Nostocales | Scytonemataceae | Scytonema | Scytonema millei |  |
| Nostocales | Stigonemataceae | unclassified Stigonemataceae | Cyanobacterium PCC 7702 |  |
| Nostocales | Tolypothrichaceae | Hassallia | Hassallia byssoidea |  |
| Nostocales | Tolypothrichaceae | Tolypothrix | Scytonema hofmanni |  |
| Nostocales | Tolypothrichaceae | Tolypothrix | Tolypothrix bouteillei |  |
| Nostocales | Tolypothrichaceae | Tolypothrix | unclassified Tolypothrix | Scytonema hofmanni UTEX B 1581 |
| Nostocales | Tolypothrichaceae | Tolypothrix | unclassified Tolypothrix | Tolypothrix sp. PCC 7910 |
| Nostocales | Tolypothrichaceae | Tolypothrix | unclassified Tolypothrix | Tolypothrix sp. NIES-4075 |
| Oscillatoriophycideae | Chroococcales | unclassified Chroococcales | Chroococcales cyanobacterium IPPAS B-1203 |  |
| Oscillatoriophycideae | Chroococcales | Aphanothecaceae | Gloeothece | Gloeothece verrucosa |
| Oscillatoriophycideae | Chroococcales | Entophysalidaceae | Chlorogloea | unclassified Chlorogloea |
| Oscillatoriophycideae | Chroococcales | Chroococcaceae | Cyanosarcina | Cyanosarcina burmensis |
| Oscillatoriophycideae | Chroococcales | Chroococcaceae | Chroogloeocystis | Chroogloeocystis siderophila |
| Oscillatoriophycideae | Chroococcales | Chroococcaceae | Gloeocapsopsis | unclassified Gloeocapsopsis |
| Oscillatoriophycideae | Chroococcales | Chroococcaceae | Gloeocapsa | unclassified Gloeocapsa |
| Oscillatoriophycideae | Oscillatoriales | Desertifilaceae | Desertifilum | unclassified Desertifilum |
| Oscillatoriophycideae | Oscillatoriales | Desertifilaceae | Desertifilum | unclassified Desertifilum |
| Oscillatoriophycideae | Oscillatoriales | Oscillatoriaceae | Oscillatoria | unclassified Oscillatoria |
| Oscillatoriophycideae | Oscillatoriales | Oscillatoriaceae | Phormidium | Phormidium ambiguum |
| Oscillatoriophycideae | Oscillatoriales | Coleofasciculaceae | Geitlerinema | unclassified Geitlerinema |
| Oscillatoriophycideae | Oscillatoriales | Microcoleaceae | Microcoleus | unclassified Microcoleus |
| Oscillatoriophycideae | Oscillatoriales | Microcoleaceae | Kamptonema | unclassified Kamptonema |
| Chroococcidiopsidales | Chroococcidiopsidaceae | Chroococcidiopsis | Chroococcidiopsis cubana |  |
| Chroococcidiopsidales | Chroococcidiopsidaceae | Aliterella | Aliterella atlantica |  |
| Chroococcidiopsidales | Chroococcidiopsidaceae | Chroococcidiopsis | unclassified Chroococcidiopsis | Chroococcidiopsis sp. TS-821 |
| Synechococcales | Merismopediaceae | Synechocystis | unclassified Synechocystis | Synechocystis sp. PCC 7509 |
| Synechococcales | Leptolyngbyaceae | Phormidesmis | Phormidesmis priestleyi |  |
| Synechococcales | Leptolyngbyaceae | Neosynechococcus | Neosynechococcus sphagnicola |  |
| Synechococcales | Leptolyngbyaceae | unclassified Leptolyngbyaceae | Leptolyngbyaceae cyanobacterium SL_7_1 |  |
| unclassified Cyanobacteria | Cyanobacteria bacterium UBA11371 |  |  |  |
| unclassified Cyanobacteria | Cyanobacteria bacterium UBA11049 |  |  |  |
| unclassified Cyanobacteria | Cyanobacteria bacterium UBA9273 |  |  |  |
| unclassified Cyanobacteria | Cyanobacteria bacterium UBA11148 |  |  |  |
