# Supplementary material for: A TonB-Like Protein, SjdR, Is Involved in the Structural Definition of the Intercellular Septa in the Heterocyst-Forming Cyanobacterium Anabaena
Source: mBio. 2021 Jun 8;12(3):e00483-21. doi: 10.1128/mBio.00483-21 (PMC8262864; doi:10.1128/mBio.00483-21)
Supplement: TABLE S1 [file mbio.00483-21-st001.docx]

**Table S1: Oligonucleotides used in this study**

| Oligonucleotide | Sequence (5’-3’) | Purpose |
| --- | --- | --- |
| sjdR-fw | GAATTCGTGTTAACCGTGATG | Cloning |
| sjdR-rv | GATATCGGAGAGGGACTAGG |  |
| tonB2-fw | ATTAATAGATCTGTTGCAGTACCTCAGGGTTG |  |
| tonB2-rv | ATTAATAGATCTGATTTACCTTGCAGCGATC |  |
| tonB4-fw | AGATCTCCATTCCTTTGAATTC |  |
| tonB4-rv | AGATCTGGAACAGCCTTTGGAA |  |
| exbB2-fw | GGATCCGGCGTTCTCCTTATTAG |  |
| exbB2-rv | GGATCCGCCGTCTTTGTTCCTCCC |  |
| 1636-fw | GGATCCGCATCATACTCGATCTCCC |  |
| 1636-rv | GGATCCGCTAGCCAATCATTTGCC |  |
| 1655-fw | GGATCCCCTTAGCCCGTTTAGATACC |  |
| 1655-rv | GGATCCCCTCAATCACTTCTGAACG |  |
| sf-gfp-fw | GATATCGGTGGTGGTGGTGTGAGCAAGGGCGAGGAG |  |
| sf-gfp-rv | CGGTACCTTACTTGTACAGCTCGTCC |  |
| sjdR-gfp-fw | GCATCGATGGTAATACTCACACTAACG |  |
| sjdR-gfp-rv | GCGATATCTTGACTAGGATTACTGTTTTG |  |
| exbB2-s.fw | CTATTTACAGCCGGTGG |  |
| exbB2-s.rv | CCAAGCCAGATGCAGTAG |  |
| pCSEL24-fw | GATACTTCGGCGATCACC |  |
| pCSV3-1fw | CTGATGCCGCATAGTTAAGCC |  |
| pCSV3-2fw | CCACGGAATGATGTCGTCGTG |  |
| sjdR-s.fw | CCAGAATTTAACACTGGTGAG | Screening |
| sjdR-s.rv | GCTCACGTCAATGCCTACC |  |
| tonB2-s.fw | GGCAATACCCACCTTACGG |  |
| tonB2-s.rv | GCGCTGTCGGACGTTATG |  |
| tonB3-s.fw | GACTAAGTTGGTGAGAATAGG |  |
| tonB3-s.rv | GGATCTGACTCTAGTTTCCC |  |
| tonB4-s.fw | GTACAATCTCAATCATTCTGG |  |
| tonB4-s.rv | CTGACGAAGTTGATCATTGC |  |
| 1636-s.fw | CCTCTGGTAGCACTTCTGG |  |
| 1636-s.rv | CTCAGTTACGCAATACAAAGC |  |
| 1655-s.fw | CCAGTTCCTATCAGCAGTCTC |  |
| 1655-s.rv | GCTCTAAACTGGCTAGACGG |  |
| rnpB-qRT.fw | GTAGGCGTTGGCGGTTG | qRT PCR |
| rnpB-qRT.rv | CACTGGACGTTATCCAGC |  |
| sjdR-qRT.fw | GCACCTGCTATTACTCCTCAGCC |  |
| sjdR-qRT.rv | CCCGCTTTGTGACTGGTCTC |  |
| nifH-qRT.fw | CGTATCCTACGACGTATTGG |  |
| nifH-qRT.rv | CGCCATCATTTCACCAGAGG |  |
| ntcA-qRT.fw | GTGGAGCAAGCACTGAAGG |  |
| ntcA-qRT.rv | CATATCTCGGTGCGCTAAGG |  |
| hepA-qRT.fw | CGCGGTGTCCGTTTATCTGG |  |
| hepA-qRT.rv | CTGAATCTAGGGCGCTGGTG |  |
