# Supplementary material for: A TonB-Like Protein, SjdR, Is Involved in the Structural Definition of the Intercellular Septa in the Heterocyst-Forming Cyanobacterium Anabaena
Source: mBio. 2021 Jun 8;12(3):e00483-21. doi: 10.1128/mBio.00483-21 (PMC8262864; doi:10.1128/mBio.00483-21)
Supplement: TABLE S4 [file mbio.00483-21-st004.docx]

**Table S4: Starvation status of cultures utilized for schizokinen uptake measurements**

| Strain  (No. of replicates) | Chl at OD_750_=1  (mg/ml) | Uptake rate  (mol Fe/l*h) at OD_750_=1 | Avg. uptake rate  ± SD  (mol Fe/l*h) |
| --- | --- | --- | --- |
| Wild-type (15) | 0.00087083 | 1.1*10^-8^ | 7±3.4*10^-9^ |
|  | 0.0007723 | 1.1*10^-8^ |  |
|  | 0.00066603 | 6.6*10^-9^ |  |
|  | 0.00095435 | 9.5*10^-9^ |  |
|  | 0.00088591 | 5.8*10^-9^ |  |
|  | 0.00097211 | 1.4*10^-8^ |  |
|  | 0.00084629 | 4.3*10^-9^ |  |
|  | 0.0009489 | 9.5*10^-9^ |  |
|  | 0.00073781 | 5.1*10^-9^ |  |
|  | 0.00087414 | 2.6*10^-9^ |  |
|  | 0.00063053 | 2.5*10^-9^ |  |
|  | 0.00079717 | 7.2*10^-9^ |  |
|  | 0.00067266 | 4.7*10^-9^ |  |
|  | 0.00095444 | 5.0*10^-9^ |  |
|  | 0.00068604 | 9.1*10^-9^ |  |
| AFS-I-*sjdR* (6) | 0.000668 | 10.0*10^-9^ | 6±2.5*10^-9^ |
|  | 0.000769 | 6.3*10^-9^ |  |
|  | 0.001005 | 8.8*10^-9^ |  |
|  | 0.000769 | 6.6*10^-9^ |  |
|  | 0.000703 | 5.3*10^-9^ |  |
|  | 0.000721 | 2.9*10^-9^ |  |
| AFS-I-*tonB3* (6) | 0.00056119 | 2.3*10^-11^ | 4.0±0.1*10^-11^ |
|  | 0.00064332 | 1.2*10^-10^ |  |
|  | 0.00125149 | -7.4*10^-11^ |  |
|  | 0.00118604 | -9.6*10^-11^ |  |
|  | 0.00109563 | 9.8*10^-11^ |  |
|  | 0.00093688 | 1.7*10^-10^ |  |
