# Supplementary material for: A TonB-Like Protein, SjdR, Is Involved in the Structural Definition of the Intercellular Septa in the Heterocyst-Forming Cyanobacterium Anabaena
Source: mBio. 2021 Jun 8;12(3):e00483-21. doi: 10.1128/mBio.00483-21 (PMC8262864; doi:10.1128/mBio.00483-21)
Supplement: TABLE S2 [file mbio.00483-21-st002.docx]

**Table S2: *Anabaena* sp. strains used in this study**

| Strain | Resistance | Genotype | Reference |
| --- | --- | --- | --- |
| WT | - |  |  |
| AFS-I-*sjdR* | Sp^R^/Sm^R^ | *alr0248*::pCSEL24 | This study |
| AFS-I-*tonB2* | Sp^R^/Sm^R^ | *all3585*::pCSV3 | This study |
| AFS-I-*tonB3* | Sp^R^/Sm^R^ | *all5036*::pCSV3 | Stevanovic, M., Hahn, A., Nicolaisen, K., Mirus, O., & Schleiff, E. (2012). Environmental Microbiology, 14, 1655–1670. |
| AFS-I-*tonB4* | Sp^R^/Sm^R^ | *alr5329*::pCSV3 | This study |
| AFS-I-*exbB2* | Sp^R^/Sm^R^ | *alr4587*::pCSV3 | This study |
| AFS-I-*1655* | Sp^R^/Sm^R^ | *alr1655*::pCSV3 | Kind gift from Leonard Fresenborg |
| AFS-I*-1636* | Sp^R^/Sm^R^ | *all1636*::pCSV3 | Kind gift from Leonard Fresenborg |
| AFS-*sjdR-gfp* | Sp^R^/Sm^R^ | *alr0248*::pCSV3-gfp | This study |
