# Supplementary material for: A TonB-Like Protein, SjdR, Is Involved in the Structural Definition of the Intercellular Septa in the Heterocyst-Forming Cyanobacterium Anabaena
Source: mBio. 2021 Jun 8;12(3):e00483-21. doi: 10.1128/mBio.00483-21 (PMC8262864; doi:10.1128/mBio.00483-21)
Supplement: TABLE S3 [file mbio.00483-21-st003.docx]

**Table S3: Plasmids used in this study**

| Plasmid | Resistance | Insert | Purpose | Reference |
| --- | --- | --- | --- | --- |
| pCSV3 | Sp^R^/Sm^R^ |  | Cloning | Valladares, A., Rodríguez, V., Camargo, S., Martínez-Noël, G. M. A., Herrero, A., & Luque, I. (2011). Journal of Bacteriology, 193, 1172–1182. |
| pCSEL24 | Sp^R^/Sm^R^ |  |  | Olmedo-Verd, E., Muro-Pastor, A. M., Flores, E., & Herrero, A. (2006). Journal of Bacteriology, 188, 6694–6699. |
| pCSEL21 | Amp^R^ | *gfp-mut2* |  | Olmedo-Verd, E., Muro-Pastor, A. M., Flores, E., & Herrero, A. (2006). Journal of Bacteriology, 188, 6694–6699. |
| pCSEL21-sf-gfp | Amp^R^ | *sf-gfp* |  | In here |
| pCSEL24-alr0248 | Sp^R^/Sm^R^ | Internal fragment of *sjdR* | Generation of single-recombinant mutants |  |
| pCSV3-all3585 | Sp^R^/Sm^R^ | Internal fragment of *tonB2* |  |  |
| pCSV3-all5036 | Sp^R^/Sm^R^ | Internal fragment of *tonB3* |  | Stevanovic, M., Hahn, A., Nicolaisen, K., Mirus, O., & Schleiff, E. (2012). Environmental Microbiology, 14, 1655–1670. |
| pCSV3-alr5329 | Sp^R^/Sm^R^ | Internal fragment of *tonB4* |  |  |
| pCSV3-alr4587 | Sp^R^/Sm^R^ | Internal fragment of *exbB2* |  |  |
| pCSV3-all1636 | Sp^R^/Sm^R^ | Internal fragment of *all1636* |  | Kind gift from L. Fresenborg |
| pCSV3-alr1655 | Sp^R^/Sm^R^ | Internal fragment of *alr1655* |  | Kind gift from L. Fresenborg |
| pCSV3-sjdR-sf-gfp | Sp^R^/Sm^R^ | *sjdR-sf-gfp* |  | In here |
